# Targeting alternative splicing by RNAi: from the differential impact on splice variants to triggering artificial pre-mRNA splicing

**DOI:** 10.1093/nar/gkaa1260

**Published:** 2021-01-06

**Authors:** Armin Fuchs, Stefan Riegler, Zahra Ayatollahi, Nicola Cavallari, Luciana E Giono, Barbara A Nimeth, Krishna V Mutanwad, Alois Schweighofer, Doris Lucyshyn, Andrea Barta, Ezequiel Petrillo, Maria Kalyna

**Affiliations:** Max Perutz Labs, Medical University of Vienna, Vienna 1030, Austria; Max Perutz Labs, Medical University of Vienna, Vienna 1030, Austria; Department of Applied Genetics and Cell Biology, University of Natural Resources and Life Sciences Vienna, Vienna 1190, Austria; Max Perutz Labs, Medical University of Vienna, Vienna 1030, Austria; Max Perutz Labs, Medical University of Vienna, Vienna 1030, Austria; Instituto de Fisiología, Biología Molecular y Neurociencias (IFIBYNE), CONICET-Universidad de Buenos Aires, C1428EHA, Buenos Aires, Argentina; Department of Applied Genetics and Cell Biology, University of Natural Resources and Life Sciences Vienna, Vienna 1190, Austria; Department of Applied Genetics and Cell Biology, University of Natural Resources and Life Sciences Vienna, Vienna 1190, Austria; Max Perutz Labs, University of Vienna, Vienna 1030, Austria; Department of Applied Genetics and Cell Biology, University of Natural Resources and Life Sciences Vienna, Vienna 1190, Austria; Max Perutz Labs, Medical University of Vienna, Vienna 1030, Austria; Max Perutz Labs, Medical University of Vienna, Vienna 1030, Austria; Instituto de Fisiología, Biología Molecular y Neurociencias (IFIBYNE), CONICET-Universidad de Buenos Aires, C1428EHA, Buenos Aires, Argentina; Department of Applied Genetics and Cell Biology, University of Natural Resources and Life Sciences Vienna, Vienna 1190, Austria

## Abstract

Alternative splicing generates multiple transcript and protein isoforms from a single gene and controls transcript intracellular localization and stability by coupling to mRNA export and nonsense-mediated mRNA decay (NMD). RNA interference (RNAi) is a potent mechanism to modulate gene expression. However, its interactions with alternative splicing are poorly understood. We used artificial microRNAs (amiRNAs, also termed shRNAmiR) to knockdown all splice variants of selected target genes in *Arabidopsis thaliana*. We found that splice variants, which vary by their protein-coding capacity, subcellular localization and sensitivity to NMD, are affected differentially by an amiRNA, although all of them contain the target site. Particular transcript isoforms escape amiRNA-mediated degradation due to their nuclear localization. The nuclear and NMD-sensitive isoforms mask RNAi action in alternatively spliced genes. Interestingly, Arabidopsis *SPL* genes, which undergo alternative splicing and are targets of miR156, are regulated in the same manner. Moreover, similar results were obtained in mammalian cells using siRNAs, indicating cross-kingdom conservation of these interactions among RNAi and splicing isoforms. Furthermore, we report that amiRNA can trigger artificial alternative splicing, thus expanding the RNAi functional repertoire. Our findings unveil novel interactions between different post-transcriptional processes in defining transcript fates and regulating gene expression.

## INTRODUCTION

Several post-transcriptional processes jointly orchestrate gene expression at the RNA level. Among them, alternative splicing, a process of differentially combining exons and introns or their parts to generate multiple mRNA isoforms from a single gene, significantly expands the transcriptomic landscape of eukaryotic cells (1,2). Up to 95% of human and 70% of plant multi-exonic genes are alternatively spliced producing from two to thousands of transcript variants per gene (3–7). Alternatively spliced transcripts differ by intracellular localization, sensitivity to different RNA degradation machineries, such as RNA interference (RNAi) and nonsense-mediated mRNA decay (NMD), and protein-coding potential (8,9). Growing evidence supports the existence of a cross-talk between different post-transcriptional processes in regulating gene expression; however, functional links between these processes and mechanisms of their interactions are not well understood.

RNAi is a highly conserved endogenous process that exploits small RNAs and in most cases negatively regulates gene expression by degradation and/or translational inhibition of target cytoplasmic mRNAs. miRNAs, a class of small (22–24 nucleotides) RNAs, function through interaction with their target sites, complementary sequences in mRNAs (10,11). Understanding RNAi mechanisms has led to the development of gene silencing technologies with a wide range of applications, from studies of individual gene functions and high throughput genetic screens to sequence-targeted precision medical therapy and crop improvement (12–17). Several RNAi approaches were developed that co-opted the endogenous pathway at different stages. Among them, artificial miRNAs (also termed shRNA-miR (18), miR-shRNA (19), shRNAmiR (20,21), ultramiR (22) or simply as shRNA (23) and noted hereafter as amiRNA) are widely used. This approach utilizes an early step in miRNA biogenesis: endogenous pri-miRNA precursors, where miRNA and miRNA* (or guide and passenger strands, respectively, in animal systems) are replaced by amiRNA and amiRNA* sequences designed to knockdown a gene of interest (18–25). First described in human/mammalian systems (26,27), gene knockdown using endogenous miRNA scaffolds has been commonly applied in metazoan and plant species. This strategy has been implemented not only to produce an individual gene knockdown but also to generate amiRNA sets targeting particular gene families, pathways or diseases (e.g. cancer), as well as genome-wide amiRNA collections for different species, such as human, mouse, rat or Arabidopsis (14,16,23,24,28,29). On the other hand, small interfering RNAs (siRNAs) are the tool of choice when working with animal cells in culture. This strategy is using an advanced step from the endogenous silencing pathway, since siRNA molecules are directly loaded on RISC upon their entrance to the cell (30–32).

Despite outstanding progress in the development of RNAi applications, it is not completely clear why designed small RNAs have such a different efficacy. Several factors have been reported to affect knockdown efficacy, such as si/sh/amiRNA design and complementarity to the target. Features of the targeted transcript, for example, its expression level, turnover rate, si/amiRNA target site sequence context, secondary structure and RNA-binding proteins preventing access of si/amiRNA to mRNA, have also been shown to affect knockdown efficacy (33–36). Importantly, many of these RNAi challenges have been addressed at the gene level without considering alternative splicing and particular features of different isoforms.

Alternative splicing can modulate RNAi activity by changing levels of miRNAs via regulation of splicing of pri-miRNAs or pre-mRNAs of proteins involved in miRNA biogenesis (8,37–40). Furthermore, alternative splicing controls sensitivity to RNAi by altering the availability of miRNA binding sites in the transcript isoforms (41,42). Here, we applied an artificial miRNA approach to investigate RNAi sensitivity of different alternative splicing isoforms of a given gene when each of them contains a target site for an amiRNA. Moreover, we validated these findings for endogenous miRNA/transcript pairs in Arabidopsis and for the use of siRNAs in mammalian cells in culture. Dissecting the fate determinants of alternatively spliced transcripts and the mechanisms of post-transcriptional regulation of gene expression holds promise for better understanding RNAi and overcoming its limitations in studies of gene functions. This report uncovers a complex interplay between RNAi and alternative splicing, mRNA isoform compartmentalization, and NMD that must be considered in studies of endogenous RNAi and in RNAi-based applications for alternatively spliced genes.

## MATERIALS AND METHODS

### Plant growth conditions

Seeds of *A. thaliana* (Col-0 background) wild-type, transgenic and mutant plants were surface sterilized and then stratified for two days in the dark at 4°C. Plants were germinated and grown under 16 h/8 h light/dark cycle at 22°C on either soil or agar plates containing half-strength germination medium (43).

### Artificial microRNA to knockdown *At-SR30*

AmiRNA individual clone targeting *At-SR30* (CSHL_011244) (pri-miRNA319a backbone) (Supplementary Table S1) was obtained from the ABRC (Arabidopsis Biological Resource Center, ‘amiRNA at TAIR.xls’ file at ftp://ftp.arabidopsis.org/home/tair/ABRC/) and is from the amiRNA library constructed to target ∼9.000 genes in *A. thaliana* (Hannon, McCombie, Martienssen, Weigel and Sachidanandam, unpublished). This construct, together with the pSoup helper plasmid (ABRC stock number CD3–1124), was introduced into *Agrobacterium tumefaciens* strain AGL1 (44), which was used to generate transgenic Arabidopsis plants.

### Design of artificial miRNAs to knockdown *At-RS31a* and *At-RS41*

The design of amiRNAs to knockdown At-*RS31a* and At-*RS41* (Supplementary Table S1) was performed essentially as described by Niemeier *et al.* (45). Briefly, amiRNA sequences were handpicked based on established criteria (25). Selected amiRNA sequences were aligned to their respective target sites using the web-based tool RNAhybrid (46). Only amiRNA:mRNA hybrids with a minimal free energy (mfe) below −30 kcal/mol were considered for further testing. Next, amiRNA candidates were aligned to the genome of *A. thaliana* using BLAST to exclude potential off-target effects. Subsequently, mature amiRNA sequences were embedded into the pri-miRNA159a backbone and folded using the web-based tool RNAfold (rna.tbi.univie.ac.at/cgi-bin/RNAfold.cgi). Only pri-amiRNA constructs which showed similar folding patterns to pri-miR159a were considered further. Molecular cloning of amiRNA constructs was based on the Easy Cloning Vector (ECV) (45), which contains the endogenous pri-miRNA159a backbone. The miRNA159a and miRNA159a* sequences were replaced by amiRNA and amiRNA* sequences in a single polymerase chain reaction (PCR) reaction using ECV as a DNA template and primers which contained amiRNA or amiRNA* sequences as well as NheI or BsrGI restriction sites, respectively (Supplementary Table S2). Next, using flanking XbaI and SacI restriction sites, pri-amiRNA constructs were transferred to the binary plant vector pGPTV, downstream of the strong constitutive 35S RNA promoter from Cauliflower mosaic virus. Finally, these constructs were introduced into *A. tumefaciens* strain AGL1 (44) and used to generate transgenic Arabidopsis plants.

### Generation of transgenic *Arabidopsis thaliana* lines

Transgenic *A. thaliana* plants were generated either by the floral dip method (47) or by crossing. Selection of transgenic plants was done on $\frac{1}{2}$ GM agar plates in the presence of 12 μg/ml Basta (dl-phosphinotricin, Duchefa Biochemicals) or 50 μg/ml kanamycin. Following selection, positive plants were transferred to soil for further growth and subsequent genotyping. Homozygous *amiR-31a-E2*/*hen1* plants were generated by crossing. Seeds of hen1–1 (Ler) and *hen1–6* (Col-0) as well as *hen1–8* (Col-0) were kindly provided by the Olivier Voinnet and Xuemei Chen laboratories, respectively. Transgenic *upf3–1* plants expressing amiR-31a-E2 amiRNA (*amiR-31a-E2*/*upf3–1*) were generated via floral dipping (47) of homozygous *upf3–1* mutant plants using the amiR-31a-E2 construct.

### Design of minigenes and transgenic plant lines to study the artificial exon skipping event

The three minigene constructs (C1-C3) to test whether the exon 2 skipping event in *At-RS31a* is triggered by the close proximity of the amiR-31a-E2 amiRNA binding site to the 3′ splice site of intron 1 were generated as follows. The insert of control construct C1 spanning *At-RS31a* exons 1–3 was produced by amplification of genomic DNA from the Arabidopsis wild type Col-0 line using primers 93 and 94 (Supplementary Table S2). Based on the construct C1, two further constructs (C2 and C3) were generated using extension overlap PCRs. In constructs C2 and C3, the original amiRNA binding site was mutated using PCR mutagenesis. For the construct C3, a functional amiRNA binding site was re-introduced further downstream in *At-RS31a* exon 2. The primers for extension overlap PCRs and cloning of the inserts in NcoI/BamHI-digested backbone from the vector pGreenII0029-35S-TL-GFP are listed in the Supplementary Table S2. The pGreenII0029–35S-TL-GFP (harboring CaMV 35S promoter-driven sGFP(S65T)) was generated from a pGreenII0029 backbone (48). The full vector sequence is available on request. Following the confirmation by sequencing, the constructs were transformed into *A. tumefaciens* strain GV3101 (pMP90, pSoup). Subsequently, the transgenic *A. thaliana* plants carrying these minigenes were generated by floral dipping (47) of the wild type Col-0 and *amiR-31a-E2* lines and selected on $\frac{1}{2}$ GM agar plates containing 50 μg/ml kanamycin. At least three independent transgenic plants were obtained per construct and background line. RNA was isolated from leaf tissue of plants grown under 16 h/8 h light/dark cycle at 23°C in soil. Total RNA isolation, and RT-PCR analysis were performed as described below.

### Design of transgenic lines overexpressing miR156 and MIM156 to modulate *At-SPL2* and *At-SPL6*

The miR156a overexpression construct was generated by amplifying the genomic DNA from Col-0 using Q5 high fidelity DNA polymerase (NEB). The primers (listed in Supplementary Table S2) contained 5′-overhangs binding to the linearized, NcoI/XhoI-digested backbone of the cloning vector pENTR™ 4. The PCR product was excised and purified from agarose gel using GeneJET Gel Extraction Kit (Thermo Fisher) and cloned into Gateway™ pENTR™ 4 by mixing the linearized vector backbone and PCR product in a 1:1 ratio using Gibson assembly (NEB), before transformation into DH10B electro-competent *Escherichia coli* cells. Plasmids containing the gene of interest were extracted using GeneJET Plasmid Miniprep Kit (Thermo Fisher) and confirmed by sequencing. Plant expression vectors were generated using the above created entry clones and the destination vector pK7WG2D (49). Recombination of the entry clone with the destination vector was done using Gateway LR Clonase ll enzyme mix. Positive colonies with the plasmid of interest were selected for spectinomycin (150 μg/ml) resistance on LB medium. Plasmids carrying the gene of interest were extracted from overnight bacterial culture using GeneJET Plasmid Miniprep Kit (Thermo Fisher) and confirmed by sequencing. Correct plasmids were transformed into *A. tumefaciens* strain GV3101 (pMP90) before transformation of plants by floral dipping (47) to Col-0. Two independent transgenic lines were used for experiments. Seeds of a line overexpressing MIM156 (50) under control of the CaMV 35S promoter were obtained from ABRC (CD3-1555).

### Transfection and splicing analyses of human cells

HeLa cells were grown in Dulbecco's modified Eagle's medium containing 10% heat-inactivated fetal bovine serum, 100 units/ml penicillin and 100 g/ml streptomycin. Cells were transfected with control (siLuc, 5′-CUUACGCUGAGUACUUCGAdTdT-3′) or *SRSF4*-targeting (siSRSF4, 5′-GGCAGGAGAAGUGACUUAUGCAGAU-3′) siRNA duplexes at a final concentration of 40 nM using Lipofectamine 2000 (Thermo Scientific) according to the manufacturer's indications. Seventy-two hours following transfection, cells were harvested, and RNA was extracted using TriPure reagent (Roche Life Science). RNA was used for cDNA synthesis with oligo-dT and MMLV-RT (Promega) according to the manufacturer's instructions. cDNAs were amplified using Taq DNA polymerase (Invitrogen) for splicing RT-PCRs and a similar mix supplemented with SYBR Green for RT-qPCR. See Supplementary Table S2 for specific primers.

### Genotyping

Genomic DNA was isolated from a single leaf as described by Edwards *et al.* (51). PCR analysis was performed using 2 μl template DNA and 0.2 U DreamTaq DNA polymerase (Thermo Fisher Scientific) per reaction. PCR mixtures were heated to 95°C for 5 min and then subjected to 35 cycles of amplification (30 s 95°C, 20 s 58°C, 60 s 72°C). In the case of *hen1–1* and *hen1–8* mutant plants, as well as *amiR-31a-E2/hen1–1* and *amiR-31a-E2/hen1–8* crosses, Cleaved Amplified Polymorphic Sequence (CAPS) assays were carried out. For *hen1–1* mutant plants and *amiR-31a-E2/hen1–1* crosses, PCR products were digested with the restriction enzyme Hpy188I for 60 min at 37°C. In the case of *hen1–8* mutant plants and *amiR-31a-E2/hen1–8 crosses*, PCR products were digested with HpaI for 60 min at 37°C. Subsequently, digested PCR products were separated on 2% agarose gels by electrophoresis. Primers are listed in Supplementary Table S2.

### Total and small RNA isolation

Total and small RNAs were isolated from 100 mg plant tissue (whole seedlings or leaves) using either a modified TRIzol protocol (52), the mirVana RNA isolation kit (Ambion) or the RNeasy Plant Mini Kit with on-column DNase I treatment (QIAGEN). Isolated RNAs were subsequently treated with TURBO DNase (Ambion) following the manufacturer's instructions.

### RT-PCR and RT-qPCR analysis

For reverse transcription (RT), 1 μg of DNA-free total RNA was reverse transcribed using Avian Myeloblastosis Virus (AMV) reverse transcriptase and oligo (dT)_15_ using the Reverse Transcription System Kit (Promega). RT-PCR analysis was performed using 2 μl of the obtained cDNA and 0.2 U Phusion DNA Polymerase (Thermo Fisher Scientific) per reaction. The PCR mixtures were heated to 98°C for 10 min and then subjected to 30–35 cycles of amplification (10 s 98°C, 20 s 58°C, 60 s 72°C). *UBQ1* was used as a loading control. RT-qPCR reactions were prepared with the GoTaq Probe qPCR Master Mix (Promega) following the manufacturer's instructions and run on a Mastercycler® ep realplex Real-time PCR System (Eppendorf). Primer efficiencies were determined by serial dilutions of the template. Raw data were analyzed using either the relative standard curve or the delta CT method and expression was normalized to the housekeeping gene *PP2AA3*. Amplicons were examined using melting curve analysis as well as gel electrophoresis. Each RT-qPCR reaction was performed using at least one exon junction primer to exclude DNA contaminations. Primers are listed in Supplementary Table S2.

### Northern blotting

Northern blotting was performed essentially as described in Park *et al.* (53) with slight modifications. Briefly, 5 μg of total RNA, including small RNAs, were separated on a 15% denaturing urea polyacrylamide gel in 0.5× TBE running buffer (44.5 mM Tris base, 44.5 mM boric acid, 10 mM EDTA) at 150 V for ∼90 min. Following electrophoresis, RNAs were transferred to a positively charged nylon membrane by wet transfer using a Mini Trans-Blot Electrophoretic Transfer Cell (Biorad) in 0.5× TBE at 100 V for 1 h at 4°C. RNAs were crosslinked to the membrane at 60°C for 90 min using *N*-ethyl-*N*′-(dimethylaminopropyl)-carbodiimide (EDC) prior to baking for 30 min at 80°C. For prehybridization, the membrane was briefly wetted in 2× SSC buffer (300 mM NaCl, 30 mM trisodium citric acid) before the addition of 10 ml Northern hybridization buffer (5× SSC, 20 mM Na_2_HPO_4_, 7% SDS. 2× Denhardt's solution) and subsequent incubation for 2 h at 60°C in a rotating oven. In parallel, [alpha-^32^P]-UTP-labeled single-stranded RNA probes complementary to amiRNAs and U6 snRNA were generated by *in vitro* transcription using the mirVana miRNA Probe Construction Kit (Ambion) and added to the hybridization buffer prior to overnight hybridization at 60°C in a rotating oven. Finally, the membrane was washed twice with 2× SSC (supplemented with 0.1% SDS) for 15 min at 60°C and exposed to a Storage Phosphor Screen (Molecular Dynamics/GE Healthcare) for signal quantitation using Typhoon laser scanner (GE Healthcare). RNA probes are listed in Supplementary Table S2.

### 5′RLM-RACE

Using the GeneRacer Kit (LifeTechnologies), a modified 5′RLM-RACE protocol was applied to detect cleaved amiRNA targets. Briefly, 1 μg of DNA-free total RNA (capped) was directly ligated to an RNA adaptor and reverse transcribed using SuperScript III following the manufacturer's instructions. The Advantage 2 PCR Enzyme system (Clontech) was used for initial and nested touchdown PCRs using adaptor- and gene-specific primers. PCR products were analyzed by agarose gel electrophoresis, gel purified, subcloned into the pGEM-T Easy vector and finally sequenced.

### Protoplast isolation and subsequent cell fractionation

Mesophyll protoplasts were isolated from three-week-old *upf3–1* mutant plants as described by Wu *et al.* (54). Subsequent cell fractions were prepared as described by Goehring *et al.* (55) with slight modifications. Briefly, 2 × 10^6^*A. thaliana* mesophyll protoplasts were resuspended in 1 ml NIB lysis buffer (10 mM MES-KOH pH 5.5, 200 mM Sucrose, 2.5 mM EDTA, 2.5 mM DTT, 0.1 mM spermine, 10 mM NaCl, 0.2% Triton X-100, 1 U/μl RNasin (Promega)) and subsequently lysed using a 25 G gauge needle (six to ten passages). Complete lysis was confirmed by light microscopy. For the total fraction, 100 μl of lysed cells were immediately resuspended in 1 ml TRIzol (Ambion) and kept on ice until the remaining fractions were processed. All subsequent steps were performed at 4°C. The lysate was pelleted for 10 min at 500 g, and 1 ml of supernatant, which represents the cytoplasmic fraction, was removed and centrifuged for another 15 min at 10.000 g. Supernatant (800 μl) was split into 100 μl aliquots, and each was resuspended in 1 ml TRIzol. In parallel, the pelleted lysate, which represents the nuclear fraction, was carefully resuspended in 4 ml NRBT (20 mM Tris−HCl pH 7.5, 25% glycerol, 2.5 mM MgCl_2_, 0,2% Triton X-100) and centrifuged at 500 g for 10 min for a total of three times. After washing, the nuclear pellet was resuspended in 500 μl NRB2 (20 mM Tris−HCl pH 7.5, 250 mM sucrose, 10 mM MgCl_2_, 0.5% Triton X-100, 5 mM β-mercaptoethanol), carefully overlaid on top of 500 μl NRB3 (20 mM Tris−HCl pH 7.5, 1.7 M sucrose, 10 mM MgCl_2_, 0.5% Triton X-100, 5 mM β -mercaptoethanol) and centrifuged at 16 000 g for 45 min. Finally, the nuclear pellet was resuspended in 1 ml TRIzol, and RNA as well as proteins of all fractions were isolated following the manufacturer's instructions. Western blot analyses (see below for details) using anti-H3 and anti-FBPase (1:5000, Agrisera) antibodies were performed to confirm purity of nuclear and cytoplasmic fractions, respectively.

### Western blotting

Isolated proteins were separated according to their mass by SDS-PAGE using 10–16% polyacrylamide gels and the Xcell SureLock Mini cell system (Life Technologies) in 1× SDS running buffer (191 mM glycine, 24.8 mM Tris-base, 3.5 mM SDS) at 25 mA per gel. Following electrophoresis, proteins were transferred to a polyvinylidene fluoride (PVDF) membrane by wet transfer using a Mini Trans-Blot Electrophoretic Transfer Cell (Biorad) in 1× western blotting buffer (191 mM glycine, 24.8 mM Tris-base, 20% methanol) at 400 mA for 1 h at 4°C. Successful protein transfer was assessed by Ponceau S staining. Next, unspecific binding sites were blocked by incubating the PVDF membrane in 5% BSA in 1× TBST (5 mM Tris−HCl, 15 mM NaCl, 0.05% Tween-20) for 60 min at room temperature. The membrane was then incubated with primary antibody (anti-H3 and anti-FBPase, 1:5000, Agrisera) on a shaking platform overnight at 4°C. After washing the membrane three times for 5 min with 1× TBST, secondary antibody (rat anti-rabbit IgG coupled to HRP, 1:10 000 in 1× TBST, Cell Signaling Technology) incubation ensued for 60 min at room temperature. Another three washing steps followed before the membrane was incubated with 2 ml ECL Western Blotting Detection Reagent (GE Healthcare) for 1 min prior to the detection of the chemiluminescent signal using CL-XPosure films and a Curix60 (AGFA) developer.

### Cycloheximide treatment

Cycloheximide treatment was performed as described previously (56). Briefly, plants were germinated and grown under 16 h/8 h light/dark cycle at 22°C on half-strength GM medium containing 0.8% agar. Three hundred milligrams of three week old plants were transferred to 5 ml liquid medium containing half strength MS salts without vitamins, 1% sucrose, pH 5.7 plus either 20 μM CHX dissolved in dimethyl sulfoxide (DMSO) (1 μl/ml) or DMSO (1 μl/ml) only. Plants were then subjected to vacuum infiltration for 10 min and incubated further for 5 h after vacuum release. Finally, plants were flash frozen in liquid nitrogen until RNA isolation.

### Statistical analysis

Statistical analyses were performed using PRISM 6.0 (GraphPad Software, La Jolla) or Excel (Microsoft Office, Microsoft). *P*-values were calculated using an unpaired, two-legged Student's *t*-test (***P < 0.001; **P < 0.01; *P < 0.05; ns, not significant). If not stated otherwise, data represent means ± standard deviation (*n* ≥ 3).

### Accession numbers

Gene accession numbers are as follows: *At-RS31a*, AT2G46610; *At-RS41*, AT5G52040; *At-SR30*, AT1G09140; *UPF3*, AT1G33980; *HEN1*, AT4G20910; SPL2, AT5G43270; SPL6, AT1G69170; *MIR156a*, AT2G25095; *UBQ1*, AT3G52590; *PP2AA3*, AT1G13320; *SRSF4*, ENSG00000116350. Arabidopsis and human SR protein gene nomenclatures are in accordance with (57,58).

## RESULTS

### Total transcript levels show only low to moderate amiRNA efficacies for alternatively spliced genes *At-RS31a, At-RS41* and *At-SR30*

**Figure 1.**
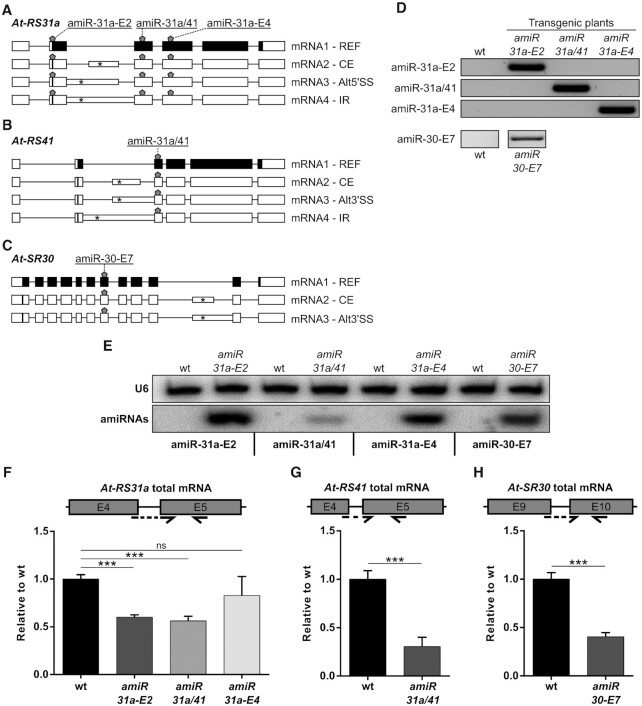
Knockdown of the SR protein genes *At-RS31a*, *At-RS41* and *At-SR30* using amiRNAs. (**A−C**) Schematics of gene structures and splicing variants of *At-RS31a* (A), *At-RS41* (B) and *At-SR30* (C) targeted by the different amiRNAs (shown by pentagons). Each amiRNA was designed to target all the splice variants of the respective gene. Canonical (reference – REF) protein-coding isoforms are called mRNA1. Other splice variants (mRNA2–4) are generated by usage of different AS events: CE, cassette exon; Alt5′/3′SS, alternative 5′/3′ splice site; IR, intron retention. Exons are shown as boxes, introns as lines. Intronic regions included due to alternative splicing are shown as narrow boxes. Positions of start codons and the first downstream premature termination codons are shown by vertical lines and asterisks within boxes, respectively. Protein-coding and non-coding regions are shown in black and white, respectively. (**D**) PCR analyses showing the detection of amiRNA constructs in the transgenic plants. Wild type (wt) plants were used as controls. (**E**) Northern blot analyses showing proper amiRNA expression in the transgenic plants. RNA probes complementary to amiR-31a-E2, amiR-31a/41, amiR-31a-E4 or amiR-30-E7 were hybridized simultaneously. U6 snRNA was used as a loading control. Wild type plants were used as controls. (**F−H**) RT-qPCR analyses of total mRNA levels for *At-RS31a* (F), *At-RS41* (G) and *At-SR30* (H) showing low knockdown efficacy in the amiRNA transgenic lines. Wild type plants were used for comparison. Primers amplify regions present in all known isoforms of the respective gene. Partial gene models are shown to visualize the analyzed regions and primer locations. Primers are shown by arrows. Dashed arrows represent primers spanning exon junctions. Expression was normalized to *PP2AA3*. Data represent means ± standard deviation (*n* ≥ 3). Student's *t*-test: ***P < 0.001; ns, not significant. Primers and RNA probes are listed in Supplementary Table S2.

We applied an amiRNA-based approach to generate knockdown lines of the *Arabidopsis thaliana* Ser/Arg-rich (SR) protein genes *At-RS31a, At-RS41* and *At-SR30* (59–61) (Figure 1A-C). All three SR genes are alternatively spliced, and alternative splicing occurs in their longest introns (Figure 1A−C). The canonical (reference – REF) mRNA1 isoforms encode the full-length reference SR proteins. Other isoforms are generated either by intron retention (IR), by usage of alternative 5′ or 3′ splice sites (Alt5′SS or Alt3′SS), or by the inclusion of a cassette exon (CE) (60–63). These splicing variants contain premature termination codons (PTCs) (Figure 1A−C), which potentially mark them for degradation *via* NMD.

To knockdown *At*-*RS31a* and *At*-*RS41*, we designed two amiRNAs that specifically target exons 2 and 4 of *At-RS31a* (amiR-31a-E2 and amiR-31a-E4) and one that targets exon 3 of both *At-RS31a* and its paralog *At-RS41* (amiR-31a/41) (Figure 1A and B, Supplementary Table S1). To knockdown *At-SR30*, we used an amiRNA, which targets exon 7 (amiR-30-E7) (Figure 1C, Supplementary Table S1). Importantly, all known splice variants of these genes contain amiRNA binding sites (Figure 1A-C). The amiRNA constructs were used to generate transgenic *A. thaliana* plants. Integration of the constructs and expression of mature amiRNAs were subsequently confirmed by genotyping and Northern blotting, respectively (Figure 1D and E).

To estimate the knockdown efficacy of the amiRNAs, we analyzed total transcript levels by reverse transcription real-time (quantitative) polymerase chain reaction (RT-qPCR) using primers that detect all transcript variants of the corresponding target gene (Figure 1F−H). This method is often used when antibodies are not available. The RT-qPCR analyses showed that *At-RS31a* total mRNA levels were down-regulated 1.7-, 1.8- and 1.2-fold in *amiR-31a-E2*, *amiR-31a/41* and *amiR-31a-E4* transgenic plants, respectively (Figure 1F). Total transcript levels of *At-RS41* and *At-SR30* decreased 3.3- and 2.5-fold in *amiR-31a/41* and *amiR-30-E7* transgenic plants, respectively (Figure 1G and H). These analyses suggest that the designed amiRNAs only moderately down-regulated *At-RS41* and *At-SR30* and exerted an even smaller effect on *At-RS31a*.

### Splice variants of a given gene are differentially affected by the same amiRNA

**Figure 2.**
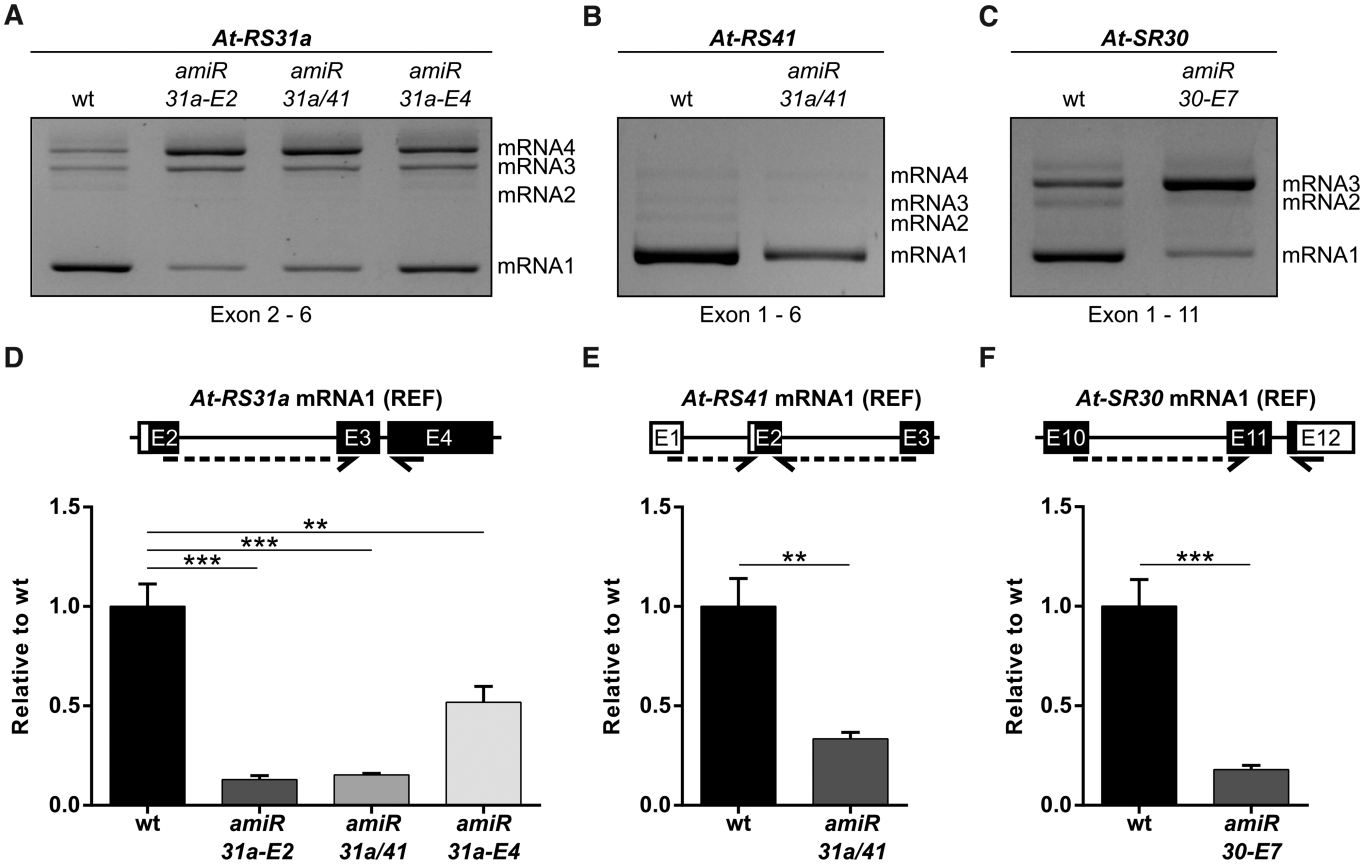
Fully spliced protein-coding transcripts are efficiently knocked down by amiRNAs. (**A−C**) Representative gel images showing differential sensitivities of the splice variants to amiRNAs. RT-PCR analyses of *At-RS31a* (A), *At-RS41* (B) and *At-SR30* (C) in amiRNA transgenic lines and wild type (wt) plants using primers that amplify all known splicing variants of the respective gene. (**D−F**) Protein-coding isoforms are effectively down-regulated by amiRNAs as shown by RT-qPCR analyses of *At-RS31a* (D), *At-RS41* (E) and *At-SR30* (F) REF mRNA1 levels in amiRNA transgenic lines when compared to wild type plants. Expression was normalized to *PP2AA3*. Primers were designed to specifically detect the respective protein-coding isoform of the gene referred to as REF mRNA1. Partial gene models are shown to visualize the analyzed regions and primer locations. Primers are shown by arrows. Dashed arrows represent primers spanning exon junctions. Primers are listed in Supplementary Table S2. Data represent means ± standard deviation (*n* ≥ 3). Student's *t*-test: ***P < 0.001; **P < 0.01; ns, not significant.

Analysis of total mRNA levels does not provide information on the abundance of individual splice variants. Since *At-RS31a*, *At-RS41* and *At-SR30* are alternatively spliced, we investigated how each isoform is affected by the amiRNAs. To this end, we performed reverse transcription polymerase chain reactions (RT-PCRs) using primers that detect all splice variants of each gene and subsequently visualized RT-PCR products by agarose gel electrophoresis (Figure 2A-C). These analyses revealed that the abundance of splice isoforms is affected to different extents by amiRNAs, even though all of them contain the respective amiRNA target sites. Importantly, only the REF mRNA1 protein-coding isoforms showed a clear down-regulation (Figure 2A−C). Some isoforms displayed increased amounts in response to the amiRNAs. However, it is necessary to consider that RT-PCR relies on relative abundances. Due to this fact, if one isoform is down-regulated in response to an amiRNA, the competitive nature of PCR may generate an observed relative increase in another one that is actually not affected at all and *vice versa*. To overcome this issue, we quantified each particular isoform by RT-qPCR with specific primers. Indeed, *At-RS31a* mRNA1 levels were down-regulated 7.8-fold (*amiR-31a-E2* line), 6.5-fold (*amiR-31a/41* line) and 1.9-fold (*amiR-31a-E4* line) (Figure 2D). This considerably exceeds the observed reduction when testing total transcript levels in the respective amiRNA lines (1.7-, 1.8- and 1.2-fold; see Figure 1F). Similarly, *At-SR30* mRNA1 abundance decreased 5.6-fold, while only 2.5-fold down-regulation was observed when testing total mRNA level (Figures 2F and 1H). By contrast, changes of *At-RS41* mRNA1 and total mRNA levels were almost identical (3- and 3.3-fold, respectively) (Figures 2E and 1G), however, in this case, the other transcript isoforms have a very low abundance (Figure 2B). Based on these results, we reasoned that the greatly reduced levels of REF mRNA1 transcripts, when compared to total transcripts, is due to the much higher abundances of alternative splicing variants in *At-RS31a* and *At-SR30* (Figure 2A and C).

**Figure 3.**
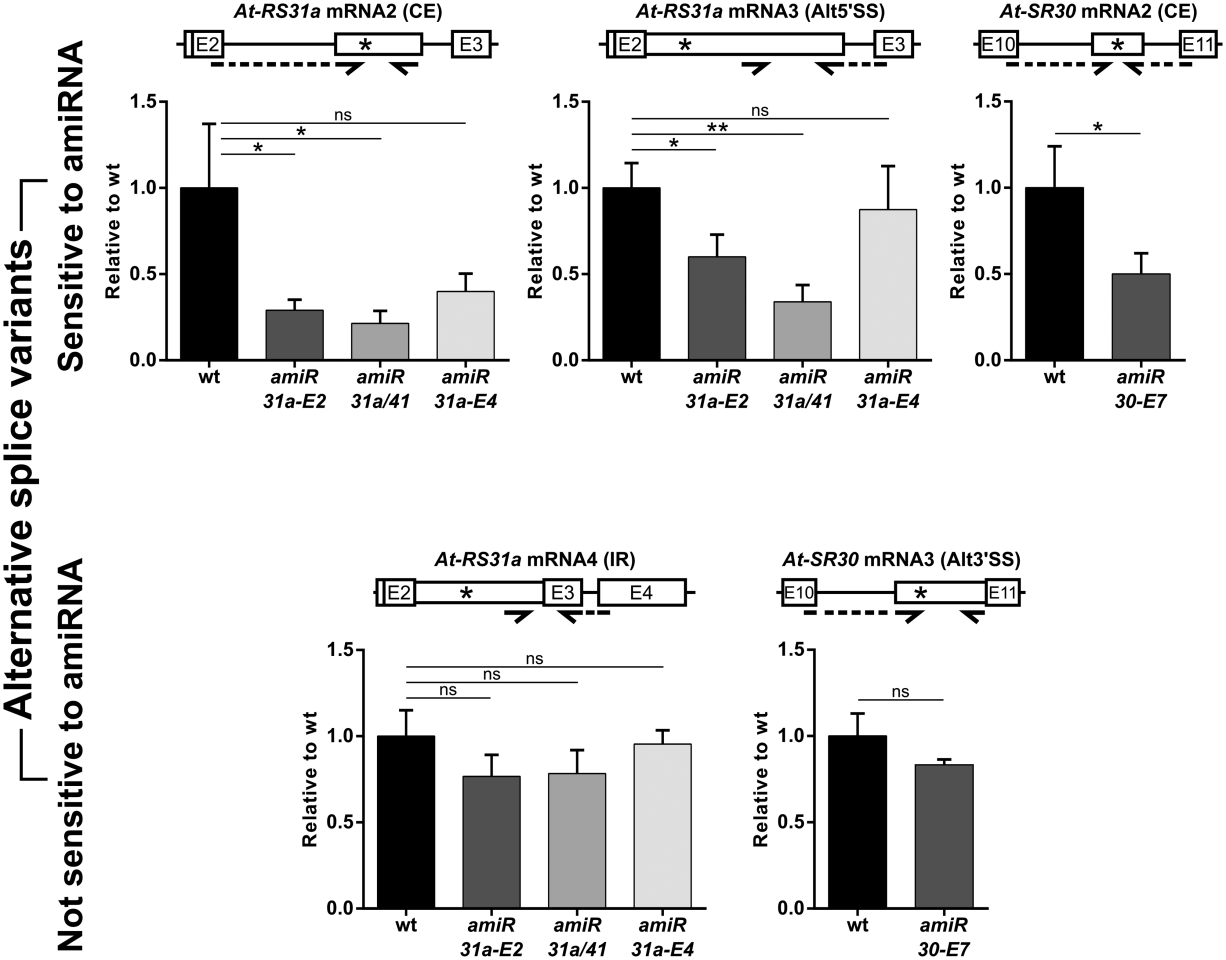
Alternative splice variants of *At-RS31a* and *At-SR30* exhibit differential sensitivities to amiRNAs. RT-qPCR analyses of *At-RS31a* and *At-SR30* splice variants in wild type (wt) and amiRNA transgenic plants showing the down-regulation exerted by the respective amiRNAs. The different splice variants were grouped depending on their amiRNA sensitivity. Sensitive splice variants, upper panel; insensitive splice variants, lower panel. Primers were designed to specifically detect the indicated mRNA isoform. Partial gene models are shown to visualize the analyzed regions and primer locations. Primers are shown by arrows. Dashed arrows represent primers spanning exon junctions. Primers are listed in Supplementary Table S2. Expression was normalized to *PP2AA3*. Data represent means ± standard deviation (*n* ≥ 3). Student's *t*-test: **P < 0.01; *P < 0.05; ns, not significant.

To confirm this, we compared levels of alternative transcripts of *At-RS31a* and *At-SR30* in amiRNA transgenic and wild type plants by RT-qPCRs using primers specific to each mRNA isoform (Figure 3). We found that At-*RS31a* CE mRNA2 and Alt5′SS mRNA3, but not I2R (intron 2 retention) mRNA4, were significantly down-regulated in *amiR-31a-E2* (3.4- and 4.7-fold) and *amiR-31a/41* (1.7- and 2.9-fold) transgenic plants, respectively. Similarly, levels of At-*SR30* CE mRNA2, but not of Alt3′SS mRNA3, decreased (2-fold) in *amiR-30-E7* transgenic plants (Figure 3). Interestingly, alternative transcripts affected by amiRNAs displayed a considerably lower level of down-regulation in comparison to the corresponding REF mRNA1 isoform (see Figure 2D−F).

These results indicate that the splice variants of a gene show different responses to the same amiRNA, although all of them contain the amiRNA target site. Furthermore, changes in total mRNA levels do not correlate with changes in the abundance of the different splice variants, especially of those encoding the full-length proteins (mRNA1 isoforms). Thus, the efficacy of amiRNAs to downregulate gene expression of alternatively spliced genes should be determined by testing the protein-coding transcripts rather than the total mRNA level of a gene.

### Transcripts targeted by the NMD machinery are less sensitive to amiRNA-mediated degradation

Our findings raise the question of why certain transcript isoforms are affected by amiRNAs to a small extent or not at all while REF mRNA1 transcripts are strongly reduced. Alternative splice variants of *At-RS31a* and *At-SR30* contain PTCs more than 50 nucleotides upstream of a splice junction and create long faux-type 3′ untranslated regions (UTRs) (Figure 1A and C), all hallmarks of NMD-sensitive (NMD^S^) transcripts. We, therefore, asked whether the reduced sensitivity of alternative isoforms to amiRNA is due to the presence of a PTC and/or to the sensitivity to NMD.

**Figure 4.**
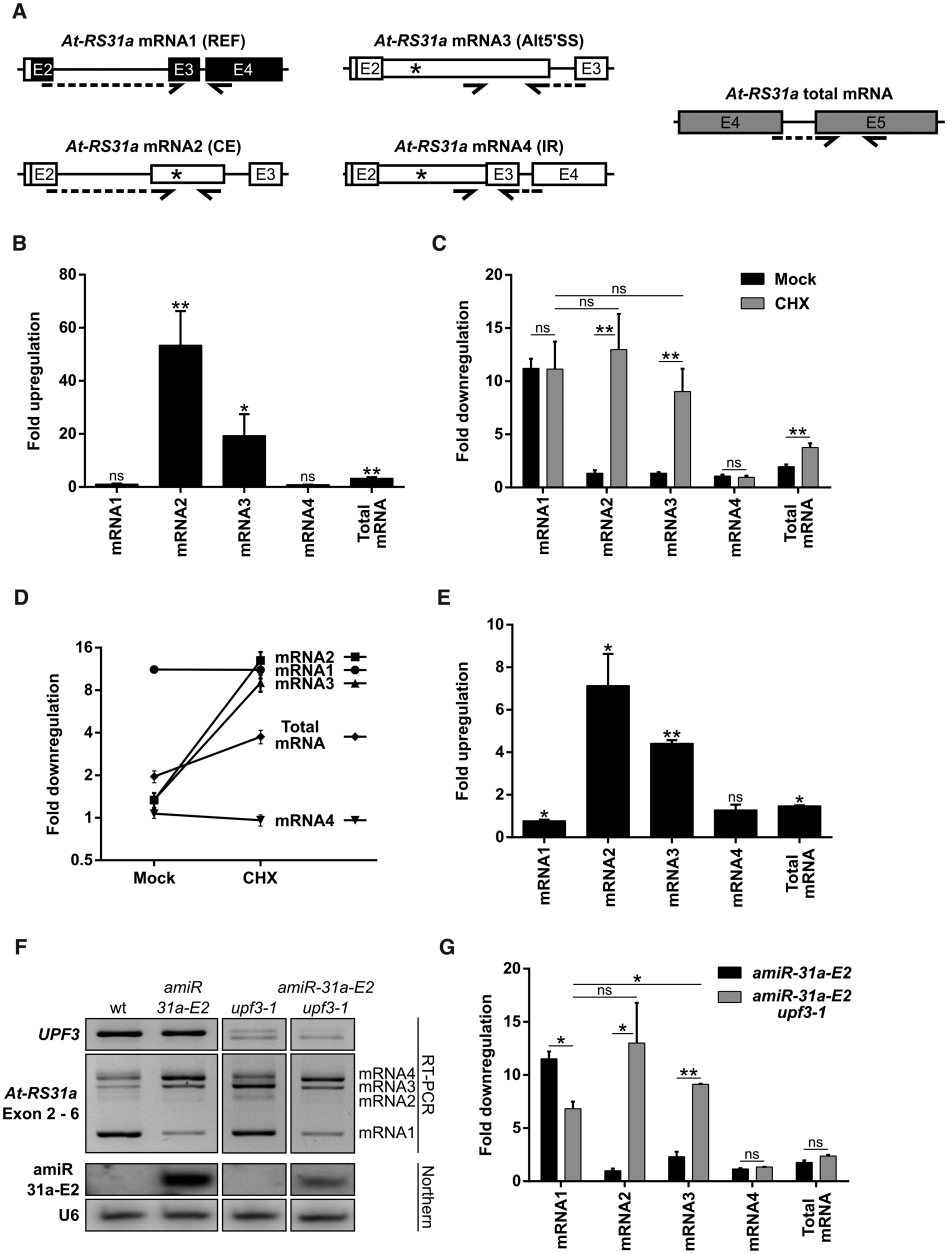
NMD masks amiRNA-mediated down-regulation of *At-RS31a* mRNA2 and mRNA3 isoforms. (**A**) Partial gene models to visualize primer locations and regions analyzed by RT-qPCRs. Primers are shown by arrows. Dashed arrows represent primers spanning exon junctions. (**B**) Up-regulation of *At-RS31a* mRNA2 and mRNA3 levels upon NMD inhibition triggered by cycloheximide (CHX) shown by RT-qPCR analyses of *At-RS31a* splice variants and total mRNA levels in CHX treated wild type plants. DMSO was used as a control. (C, D) amiRNA-mediated down-regulation of *At-RS31a* mRNA2 and mRNA3 is observed with NMD inhibition (CHX treatment). **(C)** Down-regulation of different isoforms shown by RT-qPCR analyses of *At-RS31a* splice variants and total mRNA in *amiR-31a-E2* transgenic plants treated with CHX or DMSO (mock). Wild type plants were used for standardization. **(D)** Data from (C) displayed as trends of *At-RS31a* splice isoforms in mock- or CHX-treated *amiR-31a-E2* transgenic plants. **(E)***At-RS31a* mRNA2 and mRNA3 isoforms are up-regulated in NMD-deficient mutant plants (*upf3–1*) as shown by RT-qPCR analyses of *At-RS31a* splice variants and total mRNA levels. Wild type plants were used for standardization. **(F, G)** amiRNA-mediated down-regulation of *At-RS31a* mRNA2 and mRNA3 is evidenced by NMD inhibition (*upf3–1* mutant background). **(F)** RT-PCR showing *At-RS31a* isoform abundance changes induced by the action of amiRNA and NMD deficiency in *amiR-31a-E2/upf3–1* plants. Control RT-PCR for *UPF3* expression and Northern blot analysis for mature amiRNA amiR-31a-E2 are shown. U6 snRNA was used as a loading control. **(G)** Comparison of down-regulation levels shown by RT-qPCR analyses of *At-RS31a* splice variants and total mRNA in *amiR-31a-E2* and *amiR-31a-E2/upf3–1* transgenic plants. Wild type plants were used for standardization. Expression was normalized to *PP2AA3*. Data represent means ± standard deviation *n* ≥ 3 for (B−D), and three individual transgenic lines, each analyzed in duplicates for (E, G). Student's *t*-test: **P < 0.01; *P < 0.05; ns, not significant. Primers and RNA probes are in Supplementary Table S2.

Since not every PTC-containing (PTC^+^) transcript is targeted by NMD (8,56), we first tested if these PTC^+^ isoforms are substrates of this machinery. We treated wild type plants with cycloheximide (CHX) and subsequently performed RT-qPCR analyses for all known splice variants of *At-RS31a* and *At-SR30* (Figure 4A, B and Supplementary Figure S1). NMD^S^ transcripts are expected to accumulate upon CHX treatment because CHX inhibits translation, and NMD is dependent on this process (64,65). RT-qPCR analyses showed strong up-regulation of *At-RS31a* CE mRNA2 (53.5-fold) and Alt5′SS mRNA3 (19.4-fold), as well as of *At-SR30* CE mRNA2 (7.2-fold) indicating that they are subjected to NMD (Figure 4B and Supplementary Figure S1C-E). In contrast, *At-RS31a* I2R mRNA4 and *At-SR30* Alt3′SS mRNA3 are not NMD substrates, as their levels remained unchanged upon CHX treatment (Figure 4B, Supplementary Figure S1F and S1G). Interestingly, total transcript levels of *At-RS31a* were only slightly up-regulated in response to CHX (3.3-fold) that differed markedly from the values detected for individual NMD^S^ isoforms (53.5- and 19.4-fold) (Figure 4B, Supplementary Figure S1C and S1D). These findings imply that, at the total transcript level, NMD-resistant (NMD^R^) isoforms are masking the impact of NMD on alternatively spliced genes. Thus, sensitivity to NMD needs to be determined for each transcript variant separately, as suggested earlier (56).

Next, we asked whether the sensitivity of different splicing isoforms to amiRNA-mediated degradation would change when NMD is impaired. To this end, we compared levels of alternatively spliced isoforms in wild type, *amiR-31a-E2* and *amiR-30-E7* transgenic plants treated with CHX (Figure 4C, D and Supplementary Figure S1). The extent of amiRNA-mediated down-regulation of the NMD^R^*At-RS31a* REF mRNA1 (see Figure 2A) was unaffected by CHX treatment (11.2-fold in mock-treated and 10.7-fold in CHX-treated *amiR-31a-E2* plants, compared to respectively treated wild type plants) (Figure 4C, D and Supplementary Figure S1A). In contrast, amiRNA strongly affected the levels of *At-RS31a* NMD^S^ splicing isoforms upon CHX treatment. While the effect of amiRNAs was almost negligible on NMD^S^ isoforms in mock-treated *amiR-31a-E2* plants (Supplementary Figure S1C and S1D), CHX treatment of these plants resulted in a strong down-regulation of CE mRNA2 and Alt5′SS mRNA3 (12.5- and 8.6-fold, respectively), in comparison to CHX-treated wild type plants (Figure 4C, D, Supplementary Figure S1C and S1D). Interestingly, when NMD is impaired, amiRNA-mediated down-regulation is comparable for both the PTC^+^/NMD^S^ isoforms and the PTC^−^/NMD^R^ REF mRNA1 (Figure 4C and D). The same effect is seen for the NMD^S^*At-SR30* CE mRNA2 in CHX-treated *amiR-30-E7* transgenic plants where this transcript abundance decreased 14.4-fold compared to CHX-treated wild type plants, while only a 2-fold down-regulation was observed in mock-treated *amiR-30-E7* plants (Supplementary Figure S1E). These results show that amiRNA-mediated degradation of the PTC^+^/NMD^S^ transcripts is detectable when NMD is inhibited. In contrast, CHX treatment does not change the apparent resistance to amiRNA-mediated degradation of the PTC^+^/NMD^R^ isoforms - *At-RS31a* I2R mRNA4 and *At-SR30* Alt3′SS mRNA3 (Figure 4C, D, Supplementary Figure S1F and S1G).

CHX as a general inhibitor of translation could cause pleiotropic effects. Thus, we validated our results by using *upf3–1* mutant plants impaired in NMD. RT-qPCR analyses showed that *At-RS31a* CE mRNA2 and Alt5′SS mRNA3, but not I2R mRNA4, are indeed targets of NMD (Figure 4E). Interestingly, *At-RS31a* total transcript levels increased only about 1.5-fold in *upf3–1* mutant plants (Figure 4E). To independently verify the sensitivity of the PTC^+^ isoforms to amiRNAs when NMD is impaired, we generated *amiR-31a-E2*/*upf3–1* plants (Figure 4F). Consistent with the results obtained using CHX, amiRNA-mediated degradation of PTC^+^/NMD^S^ isoforms of *At-RS31a*, CE mRNA2 and Alt5′SS mRNA3, is remarkably enhanced in NMD deficient *amiR-31a-E2*/*upf3–1* plants (Figure 4G). While the abundance of these isoforms was not significantly different in *amiR-31a-E2* and wild type plants, their levels were 12.5-fold and 9.1-fold lower in *amiR-31a-E2*/*upf3–1* than in *upf3–1* plants (Figure 4G), closely recapitulating the values observed upon CHX treatment. These values are also similar to those observed for REF mRNA1 (Figure 4C, G and Supplementary Figure S2A). As expected, PTC^+^/NMD^R^ I2R mRNA4 was not significantly affected in *amiR-31a-E2*/*upf3–1* plants (Figure 4G and Supplementary Figure S2D). Our results show that transcripts targeted by the NMD machinery appear to be less sensitive to amiRNA-mediated degradation, suggesting NMD as the primary or dominant activity.

### Splice variants can escape amiRNA-mediated degradation and NMD due to nuclear retention

Our results showed that some AS variants were not sensitive to amiRNAs (*At-RS31a* I2R mRNA4 and *At-SR30* Alt3′SS mRNA3) while others displayed only low sensitivity in the presence of NMD. In plants, the full complementarity of miRNA to mRNA usually leads to cleavage and subsequent degradation of transcripts (66). Therefore, we asked which isoforms of *At-RS31a* would be cleaved by amiRISC and whether cleavage of NMD^S^ transcripts would also occur in the presence of NMD. We took advantage of the fact that amiR-31a-E2 amiRNA targets the second exon of *At-RS31a*, just upstream of the alternative splicing events occurring in intron 2. Hence, a modified RNA-ligase-mediated Rapid Amplification of cDNA Ends (5′-RLM-RACE) analysis (from exon 3 across intron 2 to exon 2) would enable us to detect cleavage products of both fully and alternatively spliced transcripts of *At-RS31a* (Supplementary Figure S3A). Indeed, we identified cleavage products for REF mRNA1 as well as for NMD^S^ CE mRNA2 and Alt5′SS mRNA3 (Supplementary Figure S3B). In agreement with our previous results, no cleavage products for PTC^+^/NMD^R^ I2R mRNA4 were detected (Supplementary Figure S3B), suggesting that this intron-retaining splice variant escapes amiRISC-mediated cleavage. These results corroborate our findings that splice variants exhibit differential sensitivities to amiRNAs despite the presence of amiRNA target sites.

In *A. thaliana*, intron-retention splice variants usually escape NMD (56,67) due to their nuclear localization (55) as NMD operates in the cytoplasm. Similarly, plant miRISC also exert their functions of mRNA cleavage/degradation and translation inhibition in the cytoplasm. Therefore, we analyzed the intracellular localization of *At-RS31a* and *At-SR30* splice variants that are not down-regulated by amiRNA and are not sensitive to NMD. We separated nuclear and cytoplasmic fractions using cell extracts of *upf3–1* plants since NMD^S^ splice variants are often difficult to detect in wild type plant extracts. RT-PCR analyses revealed that *At-RS31a* I2R mRNA4 and *At-SR30* Alt3′SS mRNA3 were only detected in the nucleus while the remaining splice variants were also found in the cytoplasm (Supplementary Figure S3C). These results show that splice variants evade amiRNA-mediated degradation and NMD due to nuclear retention and suggest that the efficacy of amiRNAs depends on the intracellular localization of a transcript.

### Splice variants of a given gene are differentially affected by the same siRNA in human cells

**Figure 5.**
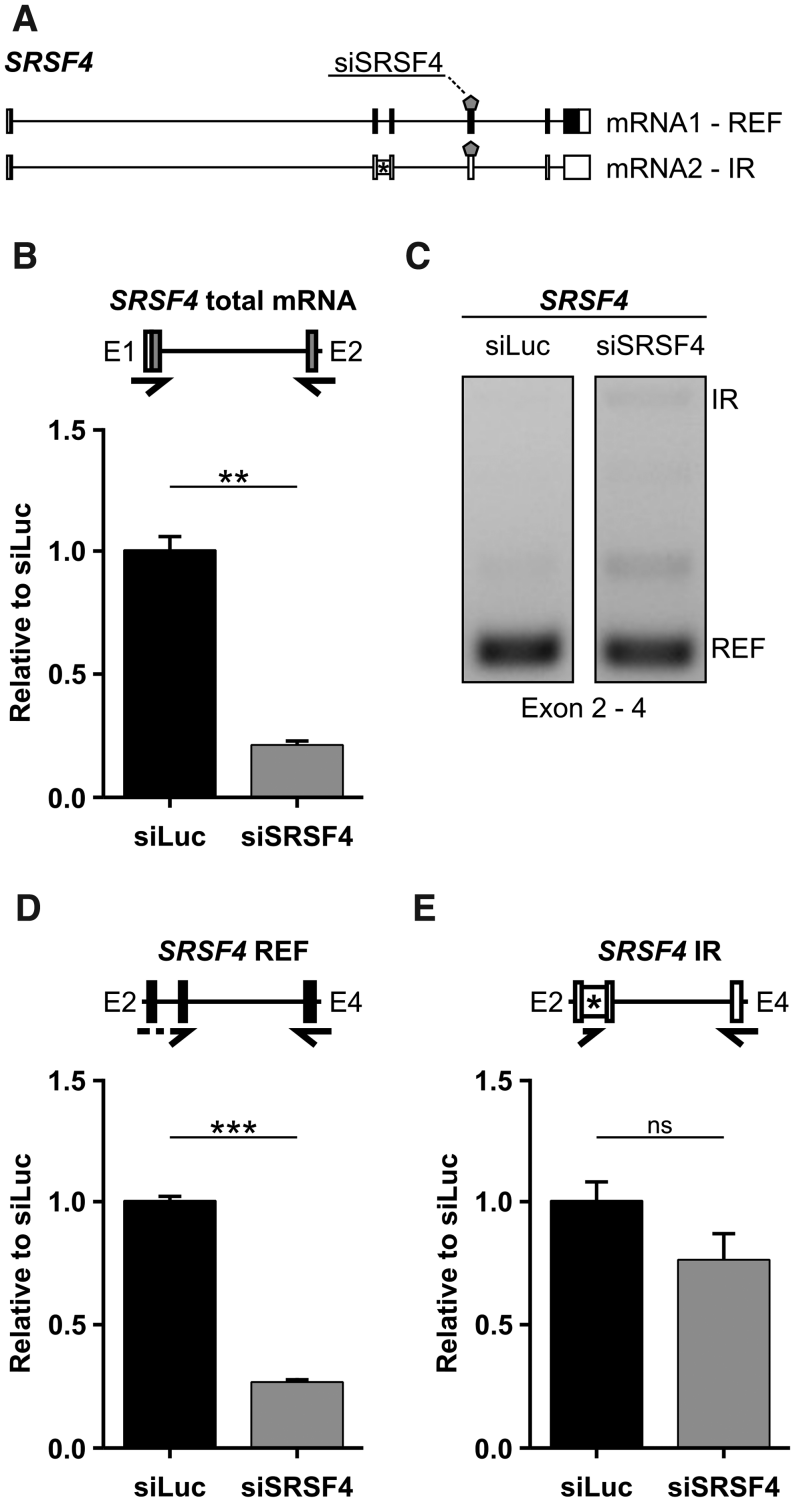
siRNA in HeLa cells differentially affects the alternative splicing isoforms of its target gene. (**A**) Gene models of *SRSF4* (*SRp75*). Intron 2 is retained in IR isoform. REF, reference transcript. siRNA binding site is shown by a pentagon (siSRSF4). (**B**) RT-PCR showing *SRSF4* isoform relative abundance changes induced by the action of siSRSF4. (C, D) Total levels of *SRSF4* are downregulated by siSRSF4 as revealed by qPCR **(C)**, REF is also downregulated (**D**) but IR isoform remains unchanged (**E**). Primers are shown by arrows. Dashed arrows represent primers spanning exon junctions. Primers are listed in Supplementary Table S2. Data represent means ± standard deviation (*n* = 3). Student's *t*-test: ***P < 0.001; **P < 0.01; ns, not significant.

We evaluated if a similar connection between RNAi and splicing outcomes is also occurring in mammals, particularly in human cells. Since our main results in plants were obtained using genes coding for SR proteins, which are alternatively spliced in different organisms, we asked whether the alternative isoforms of a human SR protein gene (*SRSF4*, Figure 5A) can be regulated in a similar manner by RNAi (siRNA). Figure 5B shows that when HeLa cells are transfected with an siRNA targeting *SRSF4*, total mRNA levels are effectively downregulated. Interestingly, when analyzing the alternative splicing pattern of *SRSF4* we observed that, although the reference isoform is clearly the most abundant, the siSRSF4 causes an increase in the relative abundance of other isoforms (Figure 5C). This could be caused by an over-accumulation of these isoforms triggered by the siRNA, by a differential sensitivity of the different isoforms to the siRNA or could be a PCR artifact. This was further investigated by RT-qPCR. While the reference transcript (REF) is efficiently downregulated by the siRNA (Figure 5D), the intron 2 retention (IR) isoform is not affected (Figure 5E). These results demonstrate that, indeed, different alternative splicing isoforms of a gene in human cells can be differentially affected by siRNA.

### An endogenous Arabidopsis miRNA affects its targets differentially

**Figure 6.**
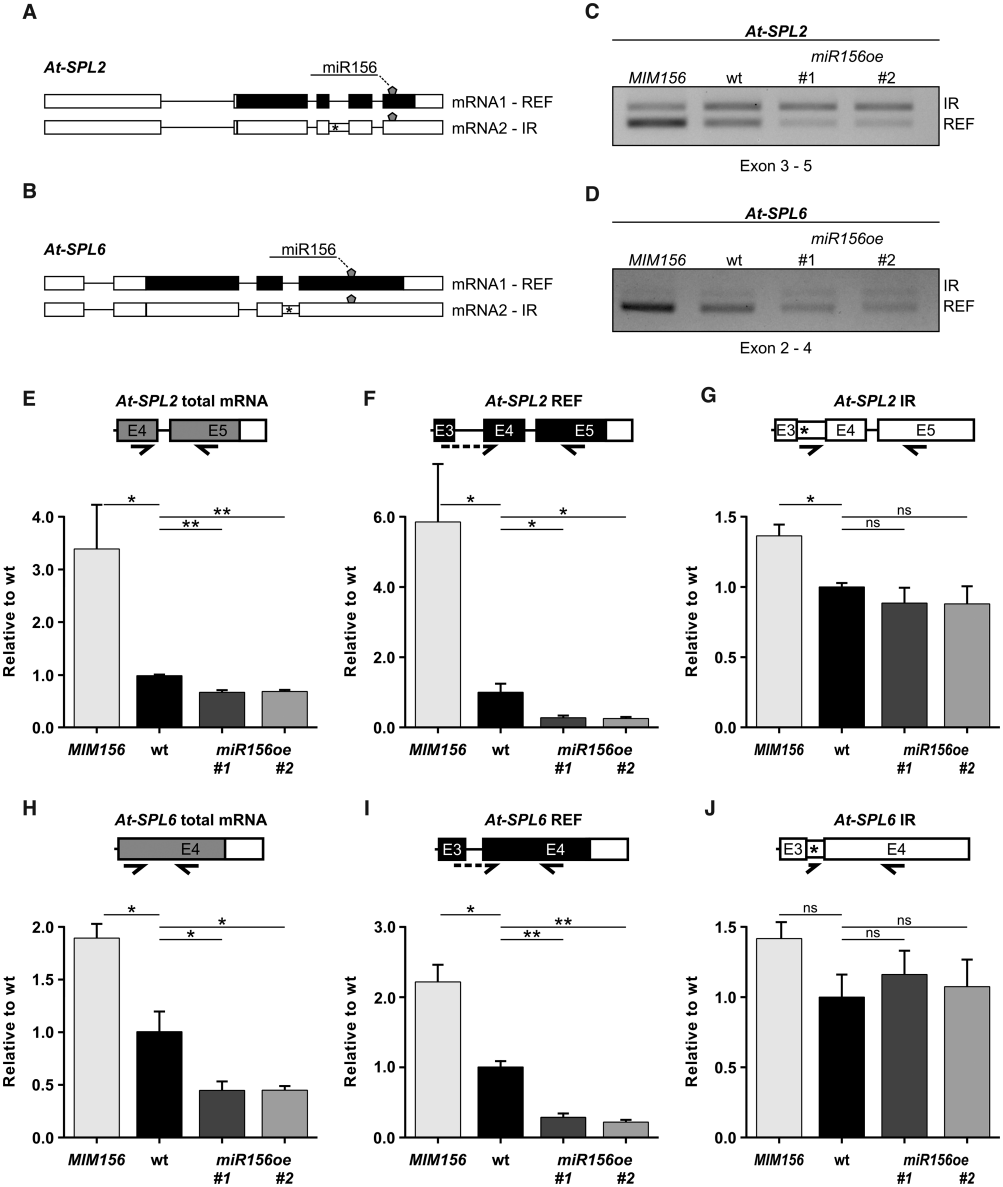
Endogenous miR156 targets SPL splicing isoforms differentially. (**A**, **B**) Gene and splicing isoform schematics of *At-SPL2* (A) and *At-SPL6* (B) are shown with the target site of miR156 depicted by a pentagon. Canonically spliced mRNA1 is termed REF, alternatively spliced transcripts, here with intron retention (IR), are labeled as mRNA2. Boxes represent exons, introns are shown as lines. Retained intronic regions are indicated by narrow boxes. Start codons and premature termination codons are illustrated by vertical lines and asterisks, respectively. Black shading indicates protein-coding regions, white represents non-coding. (**C**, **D**) RT-PCR of At-SPL2 and At-SPL6 showing changes in splicing isoform abundances due to overexpression of a miR156 target mimic (MIM156) and overexpression of miR156 (shown in two independent lines, miR156oe #1 and #2), compared to wild type. (**E−J**) RT-qPCR analyses of *At-SPL2* (E−G) and *At-SPL6* (H-J) showing total mRNA (E,H), REF isoform (F,I), and IR isoform (G,J) levels in wild type (wt), MIM156, miR156oe #1 & #2. Primers were designed to specifically amplify the indicated mRNA isoform and to span the miR156 target sequence, as represented in the partial gene models. Primers are shown by arrows, dashed arrows indicating primers spanning exon junctions. Primers are listed in Supplementary Table S2. Expression was normalized to *PP2AA3*. Data represent means ± standard deviation (*n* = 3). Student's *t*-test: **P < 0.01; *P < 0.05; ns, not significant.

Our observations of differential regulation of splicing isoforms by amiRNAs and siRNAs prompted us to investigate this phenomenon with respect to an endogenous miRNA. The SQUAMOSA PROMOTER BINDING PROTEIN-LIKE (SPL) family of transcription factors represents well studied targets of miR156 in Arabidopsis (54). Considering alternative splicing, expression level and isoform abundance, we selected *At-SPL2* and *At-SPL6* as candidate genes for further analyses. Both genes produce a protein-coding reference (REF) transcript and an intron retention (IR) isoform (Figure 6A and B). RT-PCR analyses (Figure 6C and D) demonstrated that overexpression of a target mimic for miR156 (MIM156), which essentially sequesters miR156, resulted in an increase of the reference protein-coding transcript. Conversely, overexpression of miR156 (miR156oe #1 and #2) yielded lower reference transcript levels. Neither overexpression of MIM156 nor of miR156 resulted in a substantial change of IR isoform levels. Following these initial results we performed RT-qPCRs (Figure 6E−J). Total mRNA levels of *At-SPL2* and *At-SPL6* show a ∼1.4-fold and ∼2.2-fold decrease upon miR156 overexpression (Figure 6E and H). This decrease, however, is significantly larger when observing protein-coding REF isoforms (∼3.7-fold and ∼4-fold in *At-SPL2* and *At-SPL6*, respectively; Figure 6F and I). The IR isoforms of neither gene show any significant response to overexpression of miR156 (Figure 6G and J). In response to overexpression of MIM156, mRNA levels of *At-SPL2* and *At-SPL6* generally increase. Again, this effect is stronger in REF transcripts, and the IR isoforms do not change significantly (Figure 6F, G, I and J). Collectively, our results reveal that not only amiRNAs and siRNAs regulate target transcripts differentially, but that the same holds true for an endogenous miRNA.

### An amiRNA triggers an artificial alternative splicing event

**Figure 7.**
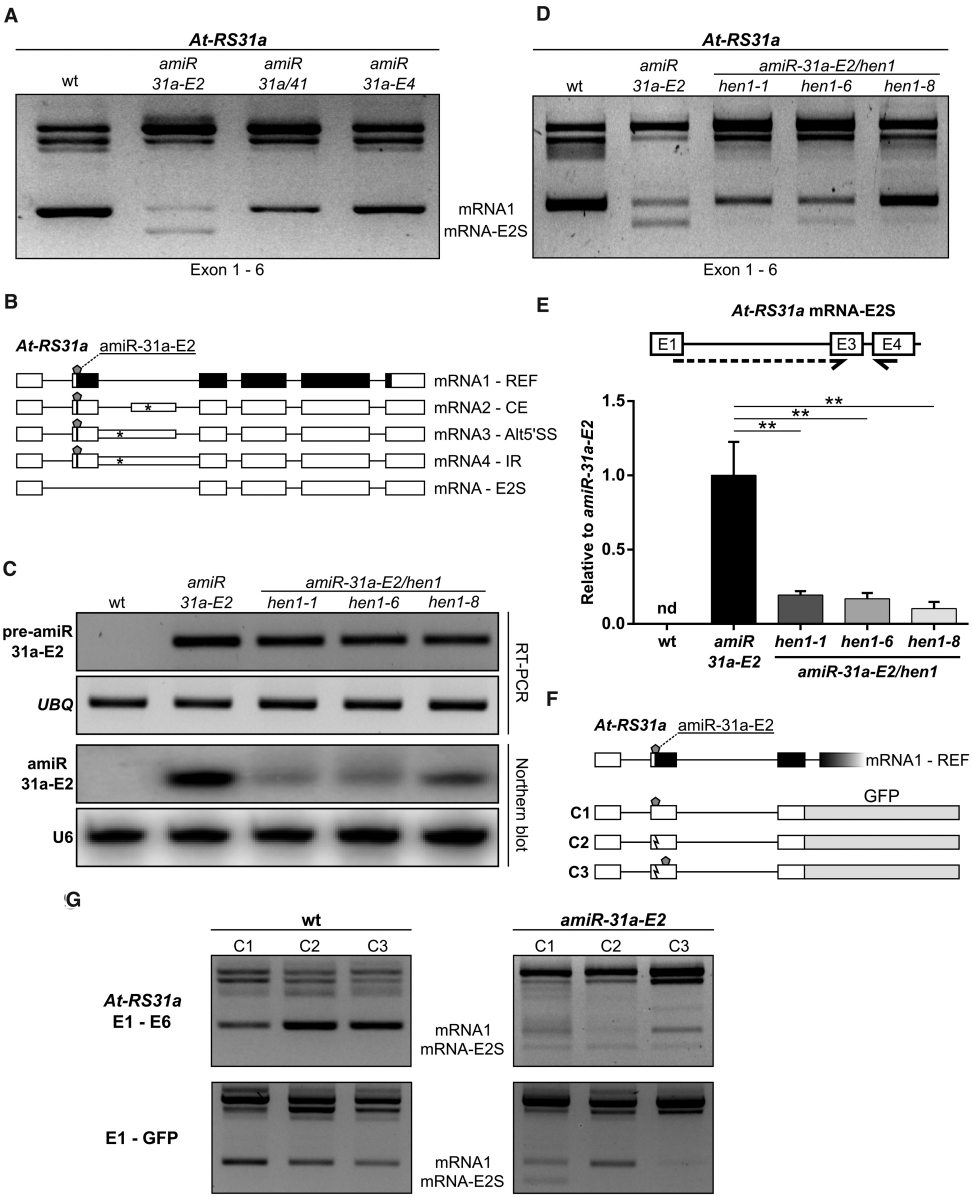
Artificial miRNA amiR-31a-E2 induces exon 2 skipping in *At-RS31a*. (**A**, **B**) The novel *At-RS31a* splice variant mRNA-E2S is generated by skipping of the second exon (E2S), which contains the amiR-31a-E2 target site. RT-PCR analysis (A) of *At-RS31a* performed using primers located in exons 1 and 6 reveals mRNA-E2S in *amiR-31a-E2* transgenic plants. (**C**) Mature amiR-31a-E2 abundance is low in the *hen1* mutant background. RT-PCR and Northern blot analyses of precursor pre-amiR-31a-E2 and mature amiRNA amiR-31a-E2, respectively, in *amiR-31a-E2*/*hen1* crosses, *amiR-31a-E2* transgenic line and wild type (wt) plants. *UBQ1* and U6 snRNA were used as loading controls for RT-PCRs and Northern blots, respectively. (D, E) The accumulation of the *At-RS31a* mRNA-E2S isoform depends on the mature amiR-31a-E2. (**D**) RT-PCR analysis of *At-RS31a* in *amiR-31a-E2*/*hen1* crosses in comparison to *amiR-31a-E2* transgenic line and wild type plants, using primers located in exon 1 and exon 6. (**E**) RT-qPCR analysis of *At-RS31a* mRNA-E2S levels in *amiR-31a-E2*/*hen1* crosses in comparison to *amiR-31a-E2* transgenic line and wild type plants. Partial gene model is shown to visualize analyzed region and primer locations. Primers are shown by arrows. Dashed arrows represent primers spanning exon junctions. Expression was normalized to *PP2AA3* and plotted relative to *amiR-31a-E2* transgenic plants since mRNA-E2S was not detectable (nd) in wild type. Data represent means ± standard deviation (*n* ≥ 3). Student's *t*-test: **P < 0.01. (F, G) The exon skipping event triggered by amiR-31a-E2 depends on the proximity of the amiRNA binding site to the exon border. **(F)** Minigene constructs corresponding to exons 1–3 of *At-RS31a* fused to *GFP*. Construct C1 contains the original amiR-31a-E2 target site (pentagon) close to the border of exon 2. Mutation in the construct C2 (zigzag line) leads to loss of the amiR-31a-E2 target site and mRNAs resistant to the amiRNA. Construct C3 has this same mutation at the original amiRNA binding site but possesses a functional amiR-31a-E2 target site downstream (pentagon). (**G**) RT-PCR analysis of endogenous *At-RS31a* using primers located in exons 1 and 6 (E1–E6) and minigene expression (E1–GFP) in plants transformed with the respective minigene construct. Primers are listed in the Supplementary Table S2.

During RT-PCR analysis of *At-RS31a* in amiRNA transgenic plants using primers to the first and last exon, we noticed the presence of an additional, potentially novel splice isoform in *amiR-31a-E2* transgenic plants, which was absent in wild type and any other *At-RS31a* amiRNA transgenic line (Figure 7A). Subsequent sequencing showed that this RT-PCR product corresponded to a novel splice variant generated by skipping of the second exon of *At-RS31a* (mRNA-E2S), the very same exon that is targeted by amiR-31a-E2 (Figure 7B). Interestingly, since exon 2 contains the translation initiation codon, mRNA-E2S does not code for the protein. Skipping of exon 2 is an artificial alternative splicing event as no supporting splice junction reads were detected in 275 RNA-Seq runs for 129 different *A. thaliana* libraries (7) and RNA-Seq datasets for CHX-treated and *upf1*/*upf3* mutant plants (68).

Based on this finding, we hypothesized that amiRNA amiR-31a-E2 changes splicing of *At-RS31a* pre-mRNA. However, we could not discriminate whether exon skipping was caused by the mature amiRNA or by either the pri- or pre-amiRNA. To answer this question, we used mutants of *HEN1*. *HEN1* is the crucial methyltransferase in *A. thaliana*, which methylates miRNA and siRNA duplexes on the ribose of their last nucleotide on each strand of the duplex (69). Unmethylated miRNAs and siRNAs in *hen1* mutants are quickly uridinylated and ultimately degraded (70). We envisioned two possible outcomes in an *amiR-31a-E2*/*hen1* background: either exon 2 skipping would be reduced, which would point towards the involvement of the methylated mature amiRNA, or exon 2 skipping would remain unchanged, which would indicate that either the pri- or pre-amiRNA interfered with splicing of *At-RS31a*. We found that *At-RS31a* exon 2 skipping was almost abolished in *amiR-31a-E2*/*hen1* plants (Figure 7C–E), suggesting that exon 2 skipping of *At-RS31a* was dependent on the mature amiRNA.

It was previously shown that alternative splicing can be modulated by nuclear antisense oligonucleotides, which affect both heterochromatin formation and RNA polymerase II processivity (71). However, experiments using the same inhibitors of chromatin formation as used by the authors (71) did not significantly affect exon 2 skipping in *amiR-31a-E2* transgenic plants (data not shown). Since the amiR-31a-E2 target site is only 18 nucleotides downstream of the 3′ splice site of intron 1, we hypothesized that the amiRNA binding could interfere with the splice site recognition. To evaluate this possibility, we generated minigene constructs (Figure 7F) encompassing exons 1 to 3, where the original amiR-31a-E2 binding site is mutated and then re-introduced towards the middle of exon 2. The control construct (C1) recapitulates behavior of the endogenous gene both in wild type and *amiR-31a-E2* transgenic plants (Figure 7G). In the construct C2 in the *amiR-31a-E2* background, the absence of the amiR-31a-E2 target site leads to the abolishment of exon 2 skipping and a substantial increase in mRNA1 relative levels. Furthermore, the re-introduction of the amiR-31a-E2 binding site farther downstream (66 nucleotides from the exon border) generates the expected decrease in mRNA1 levels but does not produce any detectable exon 2 skipping (Figure 7G). Since all endogenous *At-RS31a* controls show the mRNA-E2S variant in these backgrounds, our results strongly suggest that amiRNA binding in close proximity of a splice site could interfere with its recognition and, in turn, affect splicing and alternative splicing.

In summary, we show that amiR-31a-E2 amiRNA triggers artificial splicing of *At-RS31a* pre-mRNA, which results in exon 2 skipping, demonstrating that amiRNAs can affect gene expression not only by mRNA degradation but also by changing alternative splicing itself.

## DISCUSSION

Our study reveals a complex interplay between AS, mRNA isoform compartmentalization, NMD and RNAi. We have found that splice variants of a given gene exhibit very different responses to the same amiRNA and that not all splice variants are cleaved by amiRISC, even though all of them contain the respective amiRNA target site. These particular splice variants can escape amiRNA-mediated degradation due to nuclear retention. Additionally, the cytoplasmic transcripts targeted by NMD display a low sensitivity to amiRNAs, however, when NMD is impaired, the efficiency of their degradation by amiRNAs is very similar to that of NMD insensitive protein-coding transcripts. We also report an unexpected action of a mature (a)miRNA in triggering an artificial alternative splicing event.

The determination of knockdown efficacy is a critical component of RNAi experiments. One of the major limitations in this respect is the scarcity of antibodies. Phenotypic changes can serve as an indirect readout of si/amiRNA action. However, this approach can only be used when the underlying biological pathway is known and thus cannot be applied to genes with unknown functions. Consequently, si/amiRNA efficacy is often determined by monitoring the remaining levels of the targeted mRNA by using RT-qPCR analysis. When analyzing amiRNA efficacy for *At-RS31a* and *At-SR30* using RT-qPCR, we obtained a set of findings regarding (a)miRNA-mRNA interactions, which have been neglected so far. The total mRNA level of an alternatively spliced gene reflects the sum of all splice variants but often only one isoform is protein-coding, as the majority of *A. thaliana* alternative transcripts contain PTCs which potentially mark them for degradation via NMD (72). In the case of *At-RS31a* and *At-SR30*, only the REF mRNA1 transcripts are translated to the respective SR proteins. Assessment of total transcript levels resulted in a gross underestimation of knockdown efficacy for the protein-coding REF mRNA1 (e.g. ∼1.7- of total mRNA versus ∼8–11-fold knockdown of REF mRNA1 in *At-RS31a amiR-31a-E2* line). Therefore, it is important that amiRNA-mediated knockdown efficacies of alternatively spliced genes should either be determined on the protein level if antibodies are available or on the level of protein-coding transcripts. Importantly, our findings may also contribute to a correct evaluation of endogenous miRNA performance if the target gene is alternatively spliced and should be taken into account in microarray and RNA-seq data analyses where a 1.3–1.5 fold down-regulation cut-off is often applied. Similarly, this should be considered when determining the specificity of si/(a)miRNA-mediated silencing and monitoring off-target effects by genome-wide expression profiling. In addition, protein reduction in the absence of corresponding down-regulation in mRNA levels is often taken as an indication of si/(a)miRNA effects at the translational level. Our results, however, point to the fact that this consideration must be taken with caution in case of alternatively spliced genes.

A significant discrepancy between total mRNA and protein-coding isoform levels prompted us to analyze the effects of amiRNAs on each known splice variant of *At-RS31a* and *At-SR30*. Surprisingly, we found that all other splice variants, besides the protein-coding transcript, were affected either to a small extent or not at all by amiRNAs, despite the presence of amiRNA-target sites. These splicing variants of *At-RS31a* and *At-SR30* contain hallmarks of NMD-sensitive (NMD^S^) transcripts (73,74), thus leading to experiments investigating the interplay between NMD and amiRNA-mediated silencing. Previous studies have speculated on the benefits and pitfalls of having RNAi and NMD as two mRNA surveillance mechanisms in place (75–78), but importantly, all of these studies have been performed without taking into account the differential effects of miRNA and NMD machineries on alternatively spliced transcripts. Using cycloheximide as well as NMD-impaired *upf3–1* mutant plants, we show that two out of three and one out of two PTC^+^ splice variants of *At-RS31a* and *At-SR30*, respectively, are clearly targeted by NMD. Interestingly, similar to our amiRNA-mediated silencing results, NMD effects are also underestimated or barely seen when only total mRNA levels are analyzed (e.g., ∼1.5-fold of *At-RS31a* total mRNA versus ∼4–7 fold of its PTC^+^/NMD^S^ isoforms up-regulation in *upf3–1*). This suggests that this gene would not be determined as an NMD target when using microarray or RT-qPCR analyses of total transcript levels although two splicing variants are clearly NMD-sensitive. Indeed, At-*RS31a* was not found to be regulated by NMD using whole genome tiling array analysis of *upf3–1* plants (79). In our experiments, we found that PTC^+^/NMD^S^ isoforms are cleaved by amiRNAs in the presence of NMD. More importantly, when we impair NMD, these isoforms responded to amiRNA to the same degree as protein-coding REF mRNA1. These results suggest that the NMD machinery degrades them in such a way that they never accumulate sufficiently for amiRNA action to be reliably detected by RT-qPCR. This hypothesis is consistent with previous observations that unstable transcripts are usually more difficult to silence by siRNAs, and for such transcripts with high turnover rates, the addition of a novel degrading factor (in this case, amiRNA) does not exert the same strong effect as for stable transcripts (80). The observed differential sensitivity of PTC^+^/NMD^S^ transcripts to amiRNAs (and potentially to endogenous miRNAs) has important implications as 16–25% of alternatively spliced transcripts and up to 45% of alternatively spliced genes in *A. thaliana* are regulated by NMD (56). Our results imply that PTC^+^/NMD^S^ transcripts are obscured as (a)miRNA targets since they are efficiently degraded by NMD, a property that should be considered when analyzing (a)miRNA functionality.

We have also identified examples of splice variants that escape amiRNA-mediated degradation despite the presence of amiRNA target sites like *At-RS31a* mRNA4 (intron retention) and *At-SR30* mRNA3 (usage of Alt3′SS leading to the inclusion of the majority of the tenth intron). We show that these transcripts are not accessible to RISC and NMD degradation machineries due to their nuclear localization. This result is supported by our previous finding that *A. thaliana* IR transcripts evade NMD due to their retention in the nucleus (55). Nevertheless, it does not generally indicate that every IR transcript is being retained in the nucleus and thus escapes degradation in the cytoplasm. Though the majority of *A. thaliana* IR transcripts are not NMD substrates (56,67), a subset of them is turned over by NMD, implying that they are indeed transported to the cytoplasm (56). Interestingly, re-interpretation of data on IR transcripts associated with ribosomes allowed us to distinguish two groups of IR transcripts that are transported to cytoplasm: exitron-containing transcripts (81) and transcripts that have retained introns in their UTRs. As exitron-containing transcripts have been shown to possess different features and consequently different fates than canonical IR transcripts, this observation suggests UTR IR transcripts also differ from the latter. Indeed, we previously identified an IR transcript which contains a retained intron in the 5′UTR and is turned over by NMD (56). Moreover, the gene AT1G53160 encoding transcription factor SQUAMOSA PROMOTER BINDING PROTEIN-LIKE 4 (SPL4) is regulated by miR156 whose target site is situated in the 3′UTR intron which can be either retained or removed (42,82), implicating that UTR IR transcripts can be transported to the cytoplasm and cleaved by miRNA.

Concurrently, bioinformatic analysis and whole-cell degradome sequencing data have identified miRNA binding sites and cleavage products within introns of *A. thaliana* and rice genes, leading to the assumption that plant miRNAs cleave intron-containing transcripts/pre-mRNAs in the nucleus (83). However, our results clearly show that nuclear transcripts are not cleaved by amiRNAs. It seems more likely that cleavage products identified in this report (83) originate from cytoplasmic transcripts containing partial intronic sequences due to alternative splicing, alternative transcription initiation or alternative polyadenylation. Indeed, the majority (33 out of 40) of the detected intron-miRNA pairs with predicted miRNA cleavages (83) have evidence of either alternative transcription initiation or alternative splicing within the indicated introns in Araport11 (84) and AtRTD2 (7,85) *A. thaliana* transcriptomes. Interestingly, a large proportion of these alternative splicing events are retention of UTR introns (27 out of 40). Some of these UTR IR transcripts have been characterized previously as genuine miRNA targets (42,82). These findings further support our notion that in contrast to IR transcripts where canonical introns interrupt coding regions and which are retained in the nucleus, UTR IR transcripts have a different fate and can be accessed by cytoplasmic machineries such as RISC and NMD.

Importantly, our results show that not only canonical IR transcripts can be retained in the nucleus and escape amiRNA-mediated degradation and NMD, as evidenced by *At-SR30* Alt3′SS mRNA3. *At-SR30* produces mRNA2 where the 3′SS of the cassette exon (CE) corresponds to the Alt3′SS used in mRNA3 (Figure 1C). When compared to mRNA2, mRNA3 can be interpreted as an IR transcript with an intron between the CE and the downstream exon. Thus Alt3′SS mRNA3 can potentially be recognized as incompletely spliced and retained in the nucleus. Similarly, in *At-RS31*, a paralog of *At-RS31a*, Alt3′SS mRNA3, which can also be perceived as an IR isoform with the intron between the CE (used in mRNA2) and the downstream exon, is not sensitive to NMD due to nuclear retention (56,62,86,87). Concurrently, in *At-RS31a*, Alt5′SS mRNA3, which can also be seen as containing an intron between the CE used in mRNA2 and the upstream exon (Figure 1A), is transported to the cytoplasm and sensitive to amiRNAs and NMD, in contrast to Alt3′SS mRNA3 transcripts of *At-RS31* and *At-SR30*. This implies that these seemingly similar transcripts carry as yet unknown intrinsic features that control their fate which so far eludes bioinformatics-based predictions, thus requiring experimental analyses.

An important outcome of our experiments is the finding that the efficacy of amiRNAs is dependent on the compartmentalization of splice variants and thereby access to the miRNA machinery. Intrinsic features of splice variants determine their interaction with various cellular machineries carrying out export, degradation, translation and other processes. One might expect that these interactions and consequently the fates of alternatively spliced transcripts depend on the cell type, developmental stage, and environmental conditions. In this sense, it is important to note that we used the CaMV 35S promoter, which drives ubiquitous expression of the downstream sequences. Moreover, our analyses were done using whole seedlings, masking, or diluting, effects that could take place in specific group of cells. In addition, in human and mouse cell lines, comparisons of nuclear and cytoplasmic transcriptomes have demonstrated that the compartmentalization of splicing variants is cell-specific and that IR transcripts of particular genes accumulate in the nucleus during heat stress (88,89). Interestingly, the results of using siRNAs in HeLa cells (Figure 5) suggest that also in animals, RNAi efficacy is dependent on the features of the different splice variants and, most likely, dependent on their compartmentalization.

When analyzing the regulation *At-SPL2* and *At-SPL6*, we observed that their alternative splicing isoforms are regulated by miR156 in a way that resembles the regulation of the splicing isoforms of SR coding genes by amiRNAs. Briefly, IR isoforms are resistant to the overexpression of miR156 whilst reference isoforms are effectively downregulated (Figure 6). Hence, these results strengthen and widen our findings while excluding possible auto-regulatory effects that are common in SR protein coding genes. More importantly, these results also indicate that these mechanisms are physiologically relevant and could be working as part of the endogenous miR156/*SPLs* regulatory axis. This was further validated by using a transgenic line expressing a mimic of the binding site of miR156 (MIM156) (54). While its expression stabilizes the protein-coding transcripts of both, *At-SPL2* and *At-SPL6*, and these isoforms accumulate to higher levels, the IR isoforms remain almost unaffected.

We also report a previously unknown action of an amiRNA in changing the alternative splicing profile by triggering an artificial splicing event. One of the amiRNAs we used here, amiR-31a-E2, affects the alternative splicing of *At-RS31a* by inducing exon skipping of exon 2 (E2S). Since exon 2 contains the translation initiation codon, mRNA-E2S transcript does not code for the full-length protein, implying that amiRNAs can downregulate gene expression not only by mRNA degradation but also by affecting alternative splicing. This skipping event is dependent on HEN1 methyltransferase, thus excluding pri- and pre-amiRNA-mediated effects. These results suggest that amiRNA/RNAi performs an additional cleavage-independent action in *A. thaliana* nuclei, contributing to a plethora of nuclear RNAi functions such as transcriptional gene silencing, RNA-directed DNA methylation (RdDM), and DNA repair (90).

How could this amiRNA-mediated exon 2 skipping be achieved? Previously, Allo *et al.* have shown that alternative splicing can be altered by nuclear antisense oligonucleotides, which affect both heterochromatin formation and RNA polymerase II processivity (71). However, the skipping of exon 2 upon amiR-31a-E2-mediated silencing was not significantly affected by compounds affecting chromatin state (data not shown). We hypothesized that amiRISC could directly bind to nascent pre-mRNA before spliceosome assembly, thereby effectively blocking the splicing machinery. This is based on the fact that the amiR-31a-E2 target site is only 18 nucleotides downstream of the 3′ splice site of intron 1, hence, it is possible to think that the amiRISC (or the amiRNA) might mask that splice site or interfere with a splicing factor binding within exon 2. This type of interference is exploited as a tool in a successful case of gene therapy with splice-switching antisense oligonucleotides (91). Moreover, we did not detect similar effects on alternative splicing for amiR-31a/41 and amiR-31a-E4 amiRNAs, whose target sites are located farther from the splice sites (32 and 39 nucleotides downstream of the 3′ splice sites of intron 2 and intron 3, respectively). Moving the amiR-31a-E2 binding site farther from the 3′ splice site abolished exon 2 skipping, thus supporting our hypothesis that this effect is caused by the close proximity of the amiRNA binding site to the splice site (Figure 7F and G). However, it is noteworthy that, while amiR-31a-E2 can affect splicing, it is not able to cleave its targets in the nuclear compartment. We hypothesize that the (a)miRISC is being ‘disarmed’ upon re-entry to the nucleus in *A. thaliana* and/or that certain co-factors are absent in this compartment which are necessary for amiRNA-mediated cleavage. Additional investigations are required to elucidate the underlying mechanisms of (a)miRNA actions in modulating splicing decisions.

Despite the recent advent of CRISPR/Cas9 technology (92), RNA interference, and particularly, artificial miRNA approaches (also called shRNA-miR, miR-shRNA, shRNAmiR, ultramiR or ambiguously simply as shRNA) are still powerful tools to study gene functions in different organisms and for small RNA-based therapeutic and agricultural applications (12–17,24). The prevalence of alternative splicing generating multiple transcript isoforms with different fates from a single gene raises many new questions and requires additional studies in the context of RNA interference. Our findings that artificial microRNAs differentially affect splice variants and are even able to change alternative splicing itself highlight the impact of complex interactions of different post-transcriptional processes on defining transcript fates and regulating gene expression.

## Supplementary Material

gkaa1260_Supplemental_File

## Notes

Present address: Nicola Cavallari, Institute of Science and Technology Austria, Klosterneuburg 3400, Austria.
