# Supplementary material for: Targeting alternative splicing by RNAi: from the differential impact on splice variants to triggering artificial pre-mRNA splicing
Source: Nucleic Acids Res. 2021 Jan 6;49(2):1133–51. doi: 10.1093/nar/gkaa1260 (PMC7826280; doi:10.1093/nar/gkaa1260)
Supplement: gkaa1260_Supplemental_File [file gkaa1260_supplemental_file.pdf]

Supplementary Information

Targeting alternative splicing by RNAi: from the differential impact on splice variants to triggering artificial pre-mRNA splicing

Armin Fuchs[1], Stefan Riegler[1,2], Zahra Ayatollahi[1], Nicola Cavallari[1], Luciana E. Giono[3], Barbara A. Nimeth[2], Krishna V. Mutanwad[2], Alois Schweighofer[4], Doris Lucyshyn[2], Andrea Barta[1], Ezequiel Petrillo[1, 3,*], and Maria Kalyna[2,*]

[1]Max Perutz Laboratories, Medical University of Vienna, Vienna 1030, Austria

[2] Department of Applied Genetics and Cell Biology, University of Natural Resources and Life Sciences Vienna, Vienna 1190, Austria

[3] Instituto de Fisiología, Biología Molecular y Neurociencias (IFIBYNE), CONICET-Universidad de Buenos Aires, C1428EHA, Buenos Aires, Argentina

[4]Max Perutz Labs, University of Vienna, Vienna 1030, Austria

Present Address: [Nicola Cavallari], Institute of Science and Technology Austria, Klosterneuburg 3400, Austria

* To whom correspondence should be addressed:

Dr. Maria Kalyna
Tel: +43 1 47654-94312
E-mail: mariya.kalyna@boku.ac.at

Dr. Ezequiel Petrillo
Tel: +54 11 45763386-307
E-mail: petry@fbmc.fcen.uba.ar or petry1@gmail.com

The authors wish it to be known that, in their opinion, the first two authors should be regarded as joint First Authors

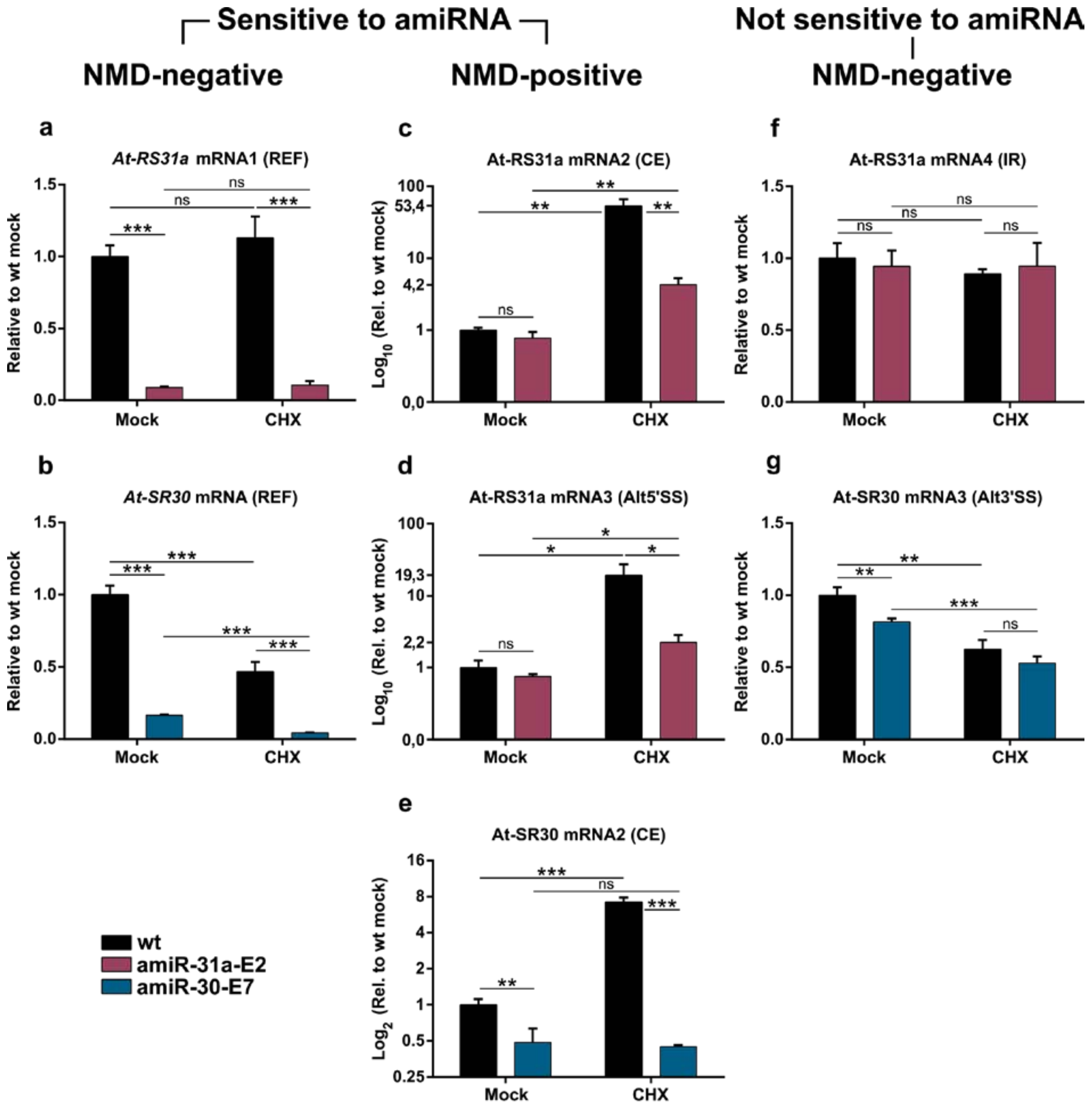

Figure S1. Differential sensitivity of *At-RS31a* and *At-SR30* splice variants to amiRNAs and nonsense-mediated mRNA decay (NMD). (A-G) Shown are RT-qPCR analyses of *At-RS31a* (A, C, D, F) and *At-SR30* (B, E, G) splice variants in mock- and cycloheximide-treated wild type, *amiR-31a-E2* (A, C, D, F) and *amiR-30-E7* (B, E, G) transgenic plants. Canonical (reference – REF) protein-coding isoforms are called mRNA1. Other splice variants (mRNA2-4) are generated by usage of different AS events: CE, cassette exon; Alt5'/3'SS, alternative 5'/3' splice site; IR, intron retention. Each splice variant was grouped depending on its amiRNA- and NMD-sensitivity. Note that *At-RS31a* mRNA2 and mRNA3 (C, D) were plotted as Log10 and *At-SR30* mRNA2 (E) as Log2. Primers were designed to specifically detect the indicated mRNA isoform (see Figures. 2 and 3 and Table S2). Expression was normalized to *PP2AA3* and plotted relative to wild type. Data represent means ± standard deviation (n ≥ 3). Student's t-test, ***p < 0.001; **p < 0.01; *p < 0.05; ns, not significant.

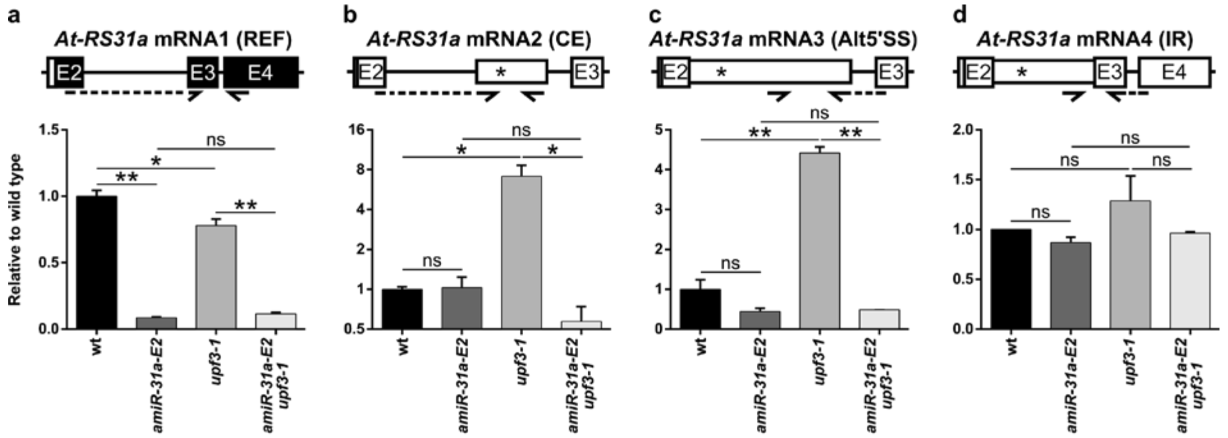

Figure S2. Mutants with NMD impairment display effective amiRNA-mediated down-regulation of *At-RS31a* mRNA2 and mRNA3, the NMD-sensitive isoforms of the gene. (A-D) RT-qPCR analyses of *At-RS31a* splice variants in wild type, *amiR-31a-E2*, *upf3-1* and *amiR-31a-E2/upf3-1* transgenic plants. Canonical (reference – REF) protein-coding isoforms are called mRNA1. Other splice variants (mRNA2-4) are generated by usage of different AS events: CE, cassette exon; Alt5'SS, alternative 5' splice site; IR, intron retention. NMD-sensitive splice variants (mRNA2 and mRNA3) show enhanced amiRNA-mediated down-regulation, while NMD-resistant mRNA1 and mRNA4 are not changing levels of down-regulation upon NMD impairment in *amiR-31a-E2/upf3-1* plants. Partial gene models are shown to visualize analyzed regions and primer locations. Primers are shown by arrows. Dashed arrows represent primers spanning exon junctions. Primers are listed in Table S2. Expression was normalized to *PP2AA3* and plotted relative to wild type. Data represent means ± standard deviation ($n \geq 3$). Student's t-test, $^{**}p < 0.01$; $^{*}p < 0.05$; ns, not significant.

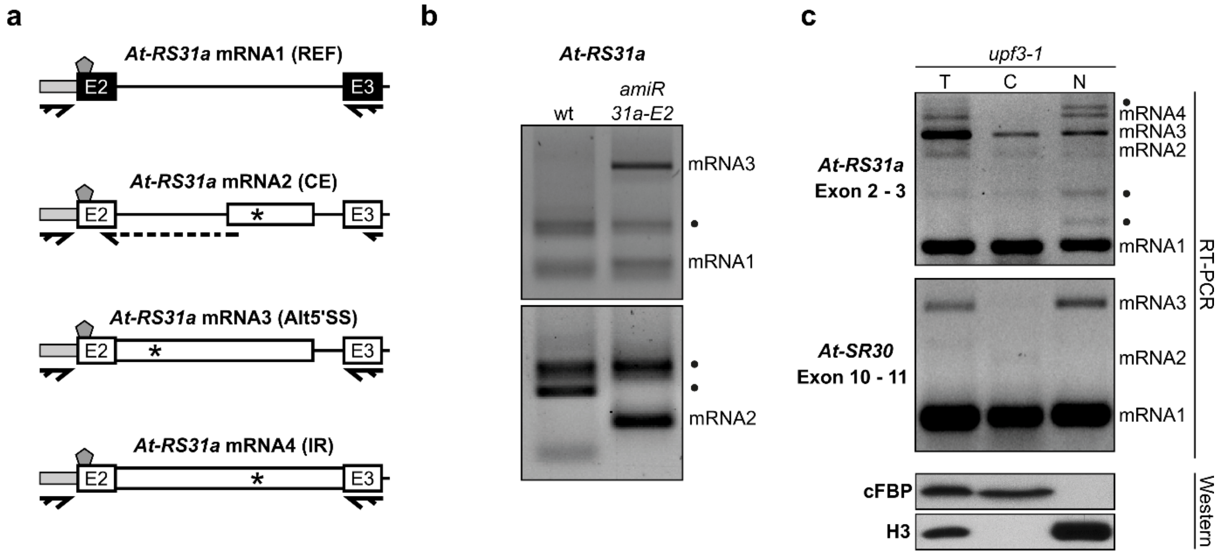

Figure S3. **Particular splice variants escape amiRNA-mediated cleavage due to nuclear retention**. **(A)** Partial gene models (cleaved exon 2 – intron 2 – exon 3) of *At-RS31a* are shown to visualize primer locations and regions analyzed by nested RT-PCRs. A modified 5'RLM-RACE was performed in wild type and *amiR-31a-E2* transgenic plants to detect which splice variants of *At-RS31a* were cleaved by amiR-31a-E2-RISC. The RNA adaptor fused to *At-RS31a* transcripts during the modified 5'RLM-RACE procedure is shown as a grey box. The amiRNA-target site is indicated as a pentagon. Positions of the first premature termination codon downstream of the translation start sites are shown by asterisks. Double-arrows and dashed arrows represent nested and exon junction primers, respectively (Table S2). **(B)** AmiRNA-induced mRNA cleavage shown by nested RT-PCR analyses of *At-RS31a* cleavage products in *amiR-31a-E2* transgenic plants. **(C)** Subcellular distribution of *At-RS31a* and *At-SR30* mRNA isoforms correlates with their sensitivity to the amiRNA. RT-PCR analyses of *At-RS31a* and *At-SR30* splice variants in cellular fractions of *upf3-1* mutant plants. T, total; C, cytoplasmic; N, nuclear. Purity of cellular fractions was confirmed by Western blot analyses of cytoplasmic (cFBP, cytosolic fructose-1,6-bisphosphatase) and nuclear (H3, Histone H3) markers. Non-specific products in (B) and (C) are shown by black dots.

Table S1. Summary of amiRNAs used in this study

amiRNA	Target gene	Gene ID	Target region	amiRNA sequence	amiRNA* sequence	Complementarity	Back bone	Vector	Clone	Construct ID
amiR-31a-E2	*At-RS31a*	AT2G46610	Exon 2	ACGTACACATGTCTCATTCTT	AAGAATGAGACTAGTGTACGT	Perfect	miR159a	ECV/pGPTV	amiR-31a-E2	35S::priamiRNA(RS31a-Ex2)-nosT
amiR-31a/41	*At-RS31a*<br>*At-RS41*	AT2G46610<br>AT5G52040	Exon 3	ACGGATTGCATCTTCAGCATC	GATGCTGAAGAACCAATCCGT	Perfect to At-RS31a<br>2 mismatches to At-RS41	miR159a	ECV/pGPTV	amiR-31a/41	35S::priamiRNA(RS31a/RS41-Ex3)-nosT
amiR-31a-E4	*At-RS31a*	AT2G46610	Exon 4	AGGCCTCTGATTCGAGACTGC	GCAGTCTCGAAAGAGAGGCCT	Perfect	miR159a	ECV/pGPTV	amiR-31a-E4	35S::priamiRNA(RS31a-Ex4)-nosT
amiR-30-E7	*At-SR30*	AT1G09140	Exon 7	ATCTTAGTTGCTATAATCCGCGACCCC	GGGGTAGCGGATTATACCAACTATGAT	2 mismatches	miR319a	pAMIR	CSHL_011244	35S::priamiRNA(SR30-Ex7)-nosT

Table S2. Primers for PCRs, RT-PCR and RT-qPCR as well as RNA probes used for Northern blotting

#	Orientation	Sequence 5'-3'	Method	Gene/Purpose
1	F, E1	GTCGTCGTCGTCTTCTAGGG	RT-PCR	At-RS31a
2	F, E2	AAGTTCGGGAGAGTGAAGCG	RT-PCR	At-RS31a
3	R, E3	TGATAACTTGCGTCGCCCAT	RT-PCR	At-RS31a
4	R, E6	TGCTCTTTGAATCGGGGACC	RT-PCR	At-RS31a
5	F, E1	ACGGCCGGTACGATTTTTCA	RT-PCR	At-RS41
6	R, E11	AGCTGCGCTCGTAAACATCT	RT-PCR	At-RS41
7	F, E1	ACGTTGGGAATTTGCCTGGAGA	RT-PCR	At-SR30
8	F, E10	CTTAGTCGTTCTCGCTCGCT	RT-PCR	At-SR30
9	R, E11	GGTGAAACTGGAGAATTCGATCTTG	RT-PCR	At-SR30
10	F	GGTTGTTCGTCACTTGCCGCC	RT-PCR	UPF3
11	R	TGCTGTAGTCTTCCGGGGCACA	RT-PCR	UPF3
12	F	ACACCATCGACAATGTCAAGGCCA	RT-PCR	UBQ
13	R	CCTGCAGTTGACAGCTCTTGGGT	RT-PCR	UBQ
14	F, E4/5	CTTGATTCTACACACAACAGCAAAT	RT-qPCR	At-RS31a total mRNA
15	R, E5	TAGGACGCCTCCGATACACT	RT-qPCR	At-RS31a total mRNA
16	F, E2/3	GTTGATATGAAGTCTGGTTATG	RT-qPCR	At-RS31a mRNA1
17	R, E4	TTTCCATCACGAGGCTTCCC	RT-qPCR	At-RS31a mRNA1
18	F, E2/I2	AGTTGATATGAAGTCTGGAG	RT-qPCR	At-RS31a mRNA2
19	R, I2	TAGGCATGAGAAAATGTAGG	RT-qPCR	At-RS31a mRNA2
20	F, I2	GTATGTAAGTGTTTGTTTGG	RT-qPCR	At-RS31a mRNA3
21	R, I2/E3	CACAAAAGCATAACCTGCATC	RT-qPCR	At-RS31a mRNA3
22	F, I2	GCATTGCAATTTCTGTGGGC	RT-qPCR	At-RS31a mRNA4
23	R, E3/4	TTCACCCTGAAAGTCCTTGG	RT-qPCR	At-RS31a mRNA4
24	F, E1/3	CTCTTCAGATTCTTCAAGGTTATG	RT-qPCR	At-RS31a mRNA-E2S
25	R, E4	GACTGCCTTTCCATCACGAG	RT-qPCR	At-RS31a mRNA-E2S
26	F, E1/2	CTTCAGATTCTTCAAGGAAT	RT-qPCR	At-RS31a, amiR-31a-E2 target site
27	R, E2	TCACTCTCCCGAACTTGC	RT-qPCR	At-RS31a, amiR-31a-E2 target site
28	F, E3	TGCTTTTGTGTATTTTGAG	RT-qPCR	At-RS31a, amiR-31a/41 target site
29	R, E3/4	TTCACCCTGAAAGTCCTTGG	RT-qPCR	At-RS31a, amiR-31a/41 target site
30	F, E3/4	AGTTGAATGGGCCAAGGAC	RT-qPCR	At-RS31a, amiR-31a-E4 target site
31	R, E4	GAACATTGAGAACTTTACC	RT-qPCR	At-RS31a, amiR-31a-E4 target site
32	F, E4/5	GGATGCTACAAATTCCAGTAAG	RT-qPCR	At-RS41 total mRNA
33	R, E5	TCCACTGAGATCACCTTATCC	RT-qPCR	At-RS41 total mRNA
34	F, E2/3	TTGATATGAAAGCTGGGTTTG	RT-qPCR	At-RS41 mRNA1
35	R, E4	AACAGAGTCTTGGAAGGTC	RT-qPCR	At-RS41 mRNA1
36	F, E3	GTTTGCTTTTGTCTATATGG	RT-qPCR	At-RS41, amiR-31a/41 target site
37	R, E3/4	ACCTCCACGGTCATTCTTTG	RT-qPCR	At-RS41, amiR-31a/41 target site
38	F, E9/10	AGTAGCAAGAGCAGGAGTGTG	RT-qPCR	At-SR30 total mRNA
39	R, E10	TATAGCGAGCGAGAACGAC	RT-qPCR	At-SR30 total mRNA
40	F, E10/11	ACAGCTCTGTCTCAAGGTCC	RT-qPCR	At-SR30 mRNA1
41	R, E12	GCTTGAGACGATTCGGGTAC	RT-qPCR	At-SR30 mRNA1
42	F, E10/I10	TACAGCTCTGTCTCAAGATC	RT-qPCR	At-SR30 mRNA2
43	R, I10/E11	ATTTTGATCTTGATTGGGACAG	RT-qPCR	At-SR30 mRNA2
42	F, E10/I10	TACAGCTCTGTCTCAAGATC	RT-qPCR	At-SR30 mRNA3
44	R, I10	GAGGAATCAGAGTAATCATAG	RT-qPCR	At-SR30 mRNA3
45	F, E5	ATTACCGCCTTCTGCTTCGTG	RT-qPCR	At-SR30, amiR-30-E7 target site
46	R, E8/9	TCGACTCATATTCCCTCACCC	RT-qPCR	At-SR30, amiR-30-E7 target site
47	F, E12	TAACGTGGCCAAAATGATGC	RT-qPCR	PP2AA3
48	R, E13	GTTCTCCACAACCGCTTGGT	RT-qPCR	PP2AA3
49	R, E3	GCCCATTCAACTGATAACTTGCGTCGCC	5'RLM-RACE	At-RS31a, first PCR
50	R, E3	CTACGGATTGCATCTTCAGCATCGCGC	5'RLM-RACE	At-RS31a, nested PCR
51	R, E2/I2	CATGCTCCTTTAGAGAAAGCTCCAGAC	5'RLM-RACE	At-RS31a, nested PCR
52	F	ATCGGCTAGCTGGAAGAAGAATGAGACTAGTGTACGTCATGAGTTGAGC AGGGTAAAG	PCR	Generation of amiR-31a-E2
53	R	GCTATGTACAAGAAGATGAAGAATGAGACATGTGTACGTGAAGAGTAAA AGCCATTAAAGGG	PCR	Generation of amiR-31a-E2
54	F	ATCGGCTAGCTGGAAGGATGCTGAAGAACCAATCCGTCATGAGTTGAGC AGGGTAAAG	PCR	Generation of amiR-31a/41

55	R	GCTATGTACAAGAAGATGGATGCTGAAGATGCAATCCGTGAAGAGTAAA AGCCATTAAAGGG	PCR	Generation of amiR-31a/41
56	F	ATCGGCTAGCTGGAAGGCAGTCTCGAAAGAGAGGCCTCATGAGTTGAGC AGGGTAAAG	PCR	Generation of amiR-31a-E4
57	R	GCTATGTACAAGAAGATGGCAGTCTCGAATCAGAGGCCTGAAGAGTAAA AGCCATTAAAGGG	PCR	Generation of amiR-31a-E4
58	R	TCTACCCGAGGCAGTTGCAT	PCR	pre-amiR-31a detection
59	F	CCACTATCCTTCGCAAGACCCTTCCT	PCR	Genotyping amiR-30-E7/CSHL_011244
60	R	ACCGGCGGTAAGGATCTGAGC	PCR	Genotyping amiR-30-E7/CSHL_011244
61	F	AGCAATTCCTCAAAAAGGTCC	PCR	Genotyping hen1-6/SALK_090960
62	R	GCCAAACATCCTGTTGAAAAG	PCR	Genotyping hen1-6/SALK_090960
63	F	CAGTTCAATCAATGGGCATC	PCR	CAPS assay hen1-1
64	R	CATCTTCTTTGTTCCACTCCC	PCR	CAPS assay hen1-1
65	F	TAGTCTCATCAAGTTATGTCT	PCR	CAPS assay hen1-8
66	R	GCAGAGAAGCGTGTTCAATC	PCR	CAPS assay hen1-8
67	F	ACGTACACATGTCTCATTCTTCCTGTCTC	Northern	amiR-31a-E2 RNA probe construction
68	F	ACGGATTGCATCTTCAGCATCCCTGTCTC	Northern	amiR-31a/41 RNA probe construction
69	F	AGGCCTCTGATTCGAGACTGCCCTGTCTC	Northern	amiR-31a-E4 RNA probe construction
70	F	ATCTTAGTTGCTATAATCCGCGACCCCCCTGTCTC	Northern	amiR-30-E7 RNA probe construction
71	F	TGGCCCCTGCGCAAGGATGACCTGTCTC	Northern	U6 RNA probe construction
72	F, E2	ACGGCGAGATGGCAGTTACG	RT-PCR	Human SRSF4, splicing
73	R, E4	TTGTGAGCATCTGCATAAGTC	RT-PCR	Human SRSF4, splicing
74	F, E1	TGGAGGTGGATCTGAAGAAC	RT-qPCR	Human SRSF4 total mRNA
75	R, E2	GTTCATAAACAGCATCATCTG	RT-qPCR	Human SRSF4 total mRNA
76	F, E2jE3	TTACGGTTCTGGACGCAGTG	RT-qPCR	Human SRSF4 REF mRNA
77	R, E4	TCCAAAGCTCTTTTCATATCAG	RT-qPCR	Human SRSF4 REF mRNA & IR
78	F, I2jE3	TATTTGAACCTTCCAAAGGTG	RT-qPCR	Human SRSF4 IR
79	F	TACAAAAAAGCAGGCTCCACTCTTTGTCTTCTCCAGTTAAAAC	PCR	Generation of 35S::miR156a
80	R	GCTGGGTCTAGATATCTCGACAAGAGAGACAGAGAAAG	PCR	Generation of 35S::miR156a
81	F, E3	CTGAGTTTGATGAGAAGAAACG	RT-PCR	At-SPL2
82	R, E5	TACGGGTTGGAGGTTGCTTGAGG	RT-PCR	At-SPL2
83	F, E2	GGCATAGAGTTTGCGAGGCT	RT-PCR	At-SPL6
84	R, E4	TGGTTGGGTTGGGTGATTGA	RT-PCR	At-SPL6
85	F, E4	TTTCCGATACCGAGCACAATAG	RT-qPCR	At-SPL2, total mRNA
86	F, E3-4j	ATCCTGGAAGGACATATGATG	RT-qPCR	At-SPL2, REF
87	F, I3	CATTTCATGTAGTTTTGGGG	RT-qPCR	At-SPL2, IR
88	R. E5	TACGGGTTGGAGGTTGCTTGAGG	RT-qPCR	At-SPL2, total & REF & IR
89	F, E4	GAAGACGACCACCGTACAAGT	RT-qPCR	At-SPL6, total mRNA
90	F, E3-4j	TCGCACCTCTCAAGATGTAG	RT-qPCR	At-SPL6, REF
91	F, I3	TCTCTGCCTTTTCCCTATCCA	RT-qPCR	At-SPL6, IR
92	R, E4	TGGTTGGGTTGGGTGATTGA	RT-qPCR	AT-SPL6, total & REF & IR
93	F, E1	AAACCATGGCTTTTCGGTTCATCATTATAAG	PCR	Generation of C1, C2 and C3 minigenes
94	R. E3	AAAGGATCCCTTGGCCCATTCAACTGATAAC	PCR	Generation of C1, C2 and C3 minigenes
95	F, E2	TTCTATGCGTCACGTCTATGTTGGGAATTTCGACTATGATAC	PCR	Generation of C1, C2 and C3 minigenes
96	R, E2	ACATAGACGTGACGCATAGAAGCAAAAGCAATCGAATTCCTAAC	PCR	Generation of C1, C2 and C3 minigenes
97	F, E2	AAGAATGAGACATGTGTACGTCAGCAAGTTCGGGAGAGTGAAG	PCR	Generation of C1, C2 and C3 minigenes
98	R, E2	ACGTACACATGTCTCATTCTTTGGCGAGTATCATAGTCGAAATTC	PCR	Generation of C1, C2 and C3 minigenes
99	F, E2	AAGAATGAGACATGTGTACGTGTTGATATGAAGTCTGGTAAAAC	PCR	Generation of C1, C2 and C3 minigenes
100	R, E2	ACGTACACATGTCTCATTCTTGCTGAACAATCTTTCAAGATC	PCR	Generation of C1, C2 and C3 minigenes
101	R	AGATGAACTTCAGGGTCAG	RT-PCR	GFP

F, forward; R, reverse; E, exon; I, intron; j, junction.
